# Pharmacokinetic Tuning Redirects β‐Lapachone From NQO1 Bioactivation to Vimentin‐Targeted Oxidation in Cancer

**DOI:** 10.1002/advs.78747

**Published:** 2026-11-09

**Authors:** Lei Dou, Huiqin Liu, Kaixin Wang, Yi Xia, Zhidong Chen, Feng Qian

**Affiliations:** ^1^ School of Pharmaceutical Sciences Beijing Frontier Research Center for Biological Structure and Key Laboratory of Bioorganic Phosphorus Chemistry & Chemical Biology (Ministry of Education) Tsinghua University Beijing People's Republic of China

**Keywords:** anti‐cancer molecule, ferroptosis, pharmacokinetics, pharmacological mechanism, vimentin, β‐lapachone

## Abstract

The “single molecule, multi‐target” paradigm is increasingly recognized among bioactive small molecules, yet the role of pharmacokinetics as a determinant of mechanism selectivity remains unexplored. Here, we demonstrate that dosing schedule governs the dominant cell death mechanism of β‐lapachone (β‐lap), an *ortho*‐naphthoquinone antitumor natural product, switching it from NQO1‐bioactivation to targeted oxidative modification of vimentin (VIM), a type III intermediate filament protein overexpressed in aggressive cancers. Using a photoaffinity probe and mass spectrometry‐based proteomics, we identified VIM as a direct binding target driving the dominant cell death mechanism under sustained low‐concentration exposure. Vimentin directly binds β‐lap with a dissociation constant at micromolar levels, and VIM essentiality for β‐lap cytotoxicity was established via knockdown and overexpression in cancer cell lines. Mechanistically, β‐lap selectively oxidizes the C328 residue of VIM, triggering disulfide crosslinking of VIM filaments, proteasomal overload, and ferroptotic cell death, a cascade independent of NQO1. A sustained‐release formulation of β‐lap achieves tumor suppression comparable to gemcitabine at its maximum tolerated dose in MIA Paca‐2 xenograft models. Taken together, these findings establish pharmacokinetic tuning as a programmable mechanistic switch, repurposing β‐lap as a broader‐spectrum, safer VIM‐targeted therapy for NQO1‐deficient malignancies and offering a generalizable strategy for natural product therapeutic expansion.

## Graphical abstract

Pharmacokinetic tuning acts as a programmable mechanistic switch for β‐lap. Sustained low‐concentration exposure redirects β‐lap from NQO1 bioactivation to direct oxidation of vimentin, driving disulfide crosslinking, proteasomal overload and ferroptosis. The sustained treatment also expands the therapeutic window by enabling efficacy at lower concentrations. These benefits provide new insight for indication and patient selection in clinical translation of β‐lap.

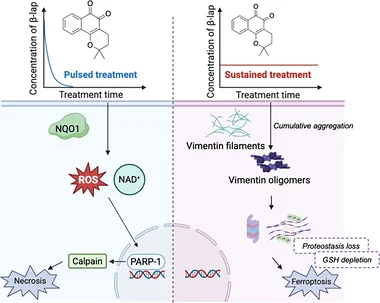

## 1 Introduction

β‐lap is an *ortho*‐naphthoquinone natural compound extracted from the lapacho tree (*Tabebuia avellanedae*) in South America, and exhibits significant therapeutic anti‐tumor activity across various cancer cells [1, 2, 3, 4]. β‐lap is known to be bioactivated by NAD(P)H: quinone oxidoreductase 1 (NQO1) [3, 5, 6], an enzyme that detoxifies most quinones through a two‐electron reduction reaction [7]. In 2000, Boothman and colleagues, based on structural similarities between β‑lap and other naphthoquinones such as menadione (vitamin K_3_), proposed that NQO1 might participate in the β‐lap bioactivation and serve as a major determinant of its cytotoxicity. Subsequent studies confirmed that the primary intracellular mechanism of β‑lap is NQO1‐dependent. β‐lap is reduced to an unstable hydroquinone via a semiquinone intermediate, which then spontaneously reacts with oxygen to regenerate β‐lap and produce two molecules of free radicals [8] (Figure 1A). The resulting H_2_O_2_ causes DNA single‐strand breaks, hyperactivation of poly(ADP‐ribose) polymerase‐1 (PARP‐1), and depletion of NAD^+^ and ATP, ultimately leading to programmed necrosis [2, 5, 9, 10].

**FIGURE 1:**
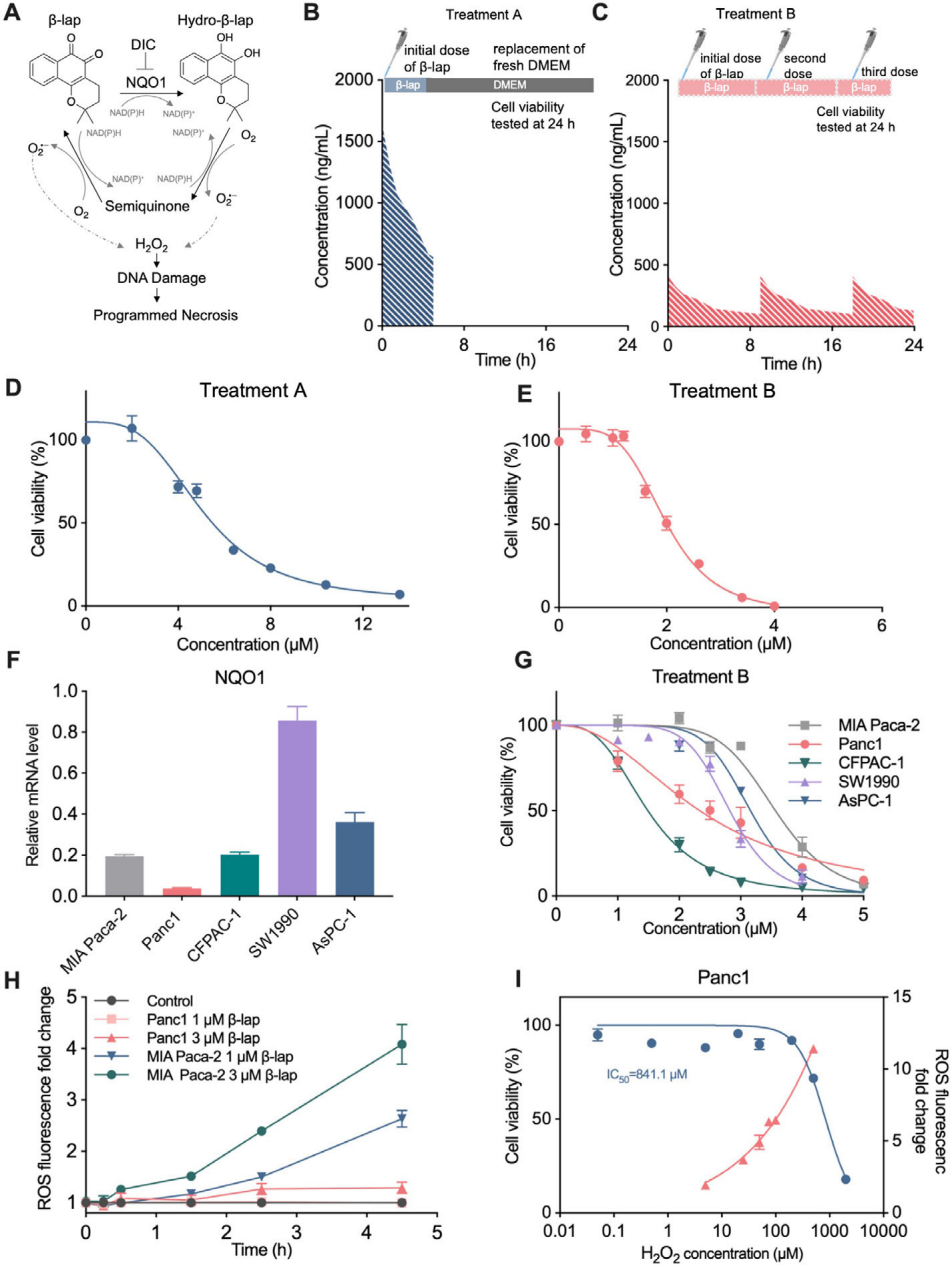
Drug exposure profile and cytotoxicity of β‐lap under two different treatments. (A) Chemical structure of β‐lap and scheme of its NQO1‐mediated bioactivation: β‐lap is reduced by NQO1 to the hydroquinone, which undergoes redox cycling in the presence of oxygen to generate a burst of ROS. (B, C), Schematic illustrations (upper panels) and corresponding concentration‐time profiles (lower panels) in KPC murine pancreatic cancer cells for (B) treatment A (initial dose of β‐lap followed by a replacement at ∼5 h with fresh medium for 19 h to rapidly eliminate the drug in the medium), (C) treatment B (repeated doses of β‐lap administered at 0, 9, and 18 h at a lower concentration range). Cell viability was assessed at 24 h. Hatched areas indicate cumulative drug exposure over time. *n* = 3. (D,E), Dose‐dependent cell viability curves following treatments A and B in KPC murine pancreatic cancer cells. *n* = 6. Data are presented as mean ± SEM. (F), Relative mRNA levels of NQO1 in five pancreatic cancer cell lines. G, Survival assays of five cell lines in (F) exposed to β‐lap (treatment B). *n* = 3. (H), The ROS levels were examined by DCFDA fluorescence at different time points after β‐lap treatment in MIA Paca‐2 cells and Panc1 cells. *n* = 3. (I), Dose‐dependent effects of H_2_O_2_ on Panc1 cell viability and intracellular ROS levels. Cell viability (blue circles, left y‐axis) and ROS fluorescence fold change (red triangles, right y‐axis) were measured following treatment with increasing concentrations of H_2_O_2_. *n* = 3.

Despite decades of research positioning β‐lap as an NQO1‐bioactivatable compound, its clinical translation has been hampered by dose‐limiting toxicities, most notably hemolytic anemia and methemoglobinemia [11, 12]. Conventional β‐lap treatments rely on high‐concentration pulsed administration to generate a rapid, robust reactive oxygen specis (ROS) burst. However, this approach causes sharply elevated peak plasma concentrations (C_max_), resulting in dose‐limiting toxicities. Various formulation approaches, including polymeric micelles, prodrug modifications, and albumin complexation have been explored to solve this problem by others and by our group [11, 13, 14, 15]. These strategies share a common assumption that if the free drug concentration in the blood is reduced, maintaining a relatively high drug exposure in the tumor can minimize toxicity while maintaining efficacy.

Inspired by this assumption, we sought to improve β‐lap's therapeutic index by engineering a sustained pharmacokinetic (PK) profile with low C_max_. We reasoned that lowering C_max_ while extending the half‐life to maintain adequate area under the curve (AUC) could reduce these adverse effects and broaden the therapeutic window. However, it remained unknown whether sustained low‐concentration exposure still generates sufficient ROS to kill tumor cells, given that β‐lap's canonical cytotoxicity depends on a rapid, NQO1‐mediated ROS burst. Nevertheless, we anticipated that the mechanism of this sustained low‐concentration treatment would remain NQO1‐dependent. Contrary to our expectation, we observed the robust antitumor activity of sustained‐release β‐lap formulation not only in NQO1‐proficient but also in NQO1‐deficient models lacking the canonical bioactivating enzyme, suggesting a previously hidden, NQO1‐independent mechanism.

This unexpected observation prompted a systematic search for the novel target and underlying mechanism of β‐lap. Identifying the specific target proteins remains central in developing mechanism‐defined therapeutics from bioactive natural products, and photoaffinity‐based pulldown has evolved into a highly valuable strategy. The method enables spatiotemporally specific capture of non‐covalent protein and small molecule interactions in native biological contexts [16, 17]. Three functional modules of a probe are combined while keeping each as small as possible: a pharmacophore that retains the bioactivity and target affinity; a photo‐crosslinking group that forms a covalent bond upon UV irradiation, thereby stabilizing transient interactions; and a terminal alkyne as a latent bioorthogonal handle [18, 19, 20]. The compact clickable handle minimizes perturbation of the parent drug's pharmacology, allowing target engagement in situ prior to ligation. Photo‐crosslinking further stabilizes weak interactions and enables detection of low abundance targets.

In this study, we designed the photoaffinity probe β‐lap‐P1, and through mass spectrometry based target identification, established vimentin (VIM) as the direct molecular target of β‐lap. Further mechanistic studies revealed that β‐lap hijacked VIM's structural plasticity through site‐specific oxidation and converted this pro‐tumor scaffold into proteotoxic aggregates that overloaded the proteasome, thereby disrupting proteostasis and ultimately triggering ferroptosis. Moreover, the sustained‐release formulation exhibited markedly improved safety compared with β‐lap formulated with hydroxypropyl‐β‐cyclodextrin (ARQ 501/761), which has completed phase I/II clinical trials [21].

Collectively, our work establishes a new paradigm: pharmacokinetic‐driven mechanism redirection transforms an NQO1‐dependent redox cycler with a restricted therapeutic window into a broadly effective, VIM‐targeted oxidant with an expanded therapeutic window, establishing formulation and dosing schedule as active determinants of molecular mechanism selectivity.

## 2 Results

### 2.1 Cytotoxicity of β‐Lap Under Two Different Treatments In Vitro

To determine whether lowering concentration and prolonging β‐lap exposure could achieve comparable antitumor effects to the conventional pulsed high‐concentration treatment of β‐lap, we used a murine KPC cell line derived from a genetically engineered mouse model of pancreatic cancer, *LSL‐Kras^G12D/^* ^+^ *; LSL‐Trp53^R172H/^* ^+^ *; Pdx‐1‐Cre* (KPC) mice. KPC cells were exposed to two treatments: (A) a single 5‑h pulse of β‐lap at gradient concentrations, followed by washout with drug‑free fresh DMEM, (B) three repeated doses of β‐lap administered at 0, 9 and 18 h at lower concentrations, designed to achieve cumulative exposure without high peak concentrations. The concentration‐time curves revealed that treatment A produced a pulsed high‐concentration exposure followed by rapid clearance, whereas treatment B maintained a lower but sustained exposure over 24 h (Figure 1B,C and Figure S1A). Notably, both treatments exhibited varying degrees of cytotoxicity in KPC cells (Figure 1D,E), demonstrating that prolonged low‐concentration exposure is an effective strategy for β‐lap mediated cell killing.

We next asked whether this sustained treatment preserved cytotoxicity in human cancer cell lines. Treatment B of β‐lap exhibited potent cytotoxicity across all five tested pancreatic cancer cell lines with different NQO1 transcription levels (Figure 1F,G). The results demonstrated that reducing the drug concentration while sufficiently extending the exposure duration enabled β‐lap to efficiently kill cancer cells. Remarkably, Panc1 cells, despite low NQO1 mRNA levels, were also sensitive to β‐lap, with an IC_50_ value of 2.3 µM, similar to that observed in NQO1‐high MIA Paca‐2 cells.

β‑Lap has been reported to induce DNA damage and programmed necrosis via ROS generation. To determine whether sustained low‐concentration β‑lap treatment also triggers ROS accumulation, we measured ROS levels over time. Consistent with previous reports, MIA Paca‑2 cells showed an approximately 4‑fold increase in ROS upon β‑lap treatment, whereas NQO1‑low Panc1 cells produced negligible ROS upon β‑lap treatment (Figure 1H). The low levels of ROS elicited by β‑lap in Panc1 cells cannot account for the observed cell death, as indicated by the concentration‐response curve of hydrogen peroxide‑induced ROS‑mediated killing (Figure 1I and Figure S1B).

### 2.2 Sustained Low‐Concentration β‐Lap Induces Cell Death in an NQO1‐Independent Manner

Since sustained low‑concentration β‑lap was found to kill NQO1‑deficient Panc1 cells without generating abundant ROS, we asked whether this treatment bypassed the classical NQO1‑dependent mechanism. To determine the role of NQO1 in treatment A‐ or B‑induced cytotoxicity, we used dicoumarol (DIC), a well‑characterized specific inhibitor of NQO1. The co‑crystal structure of DIC bound to NQO1 has revealed that DIC occupies the catalytic active site of the enzyme, thereby blocking substrate access and preventing the two‑electron reduction of quinone substrates. In the presence of DIC, β‑lap cannot be converted to its semiquinone radical intermediate, which in turn abrogates the futile redox cycle that normally generates high levels of ROS and DNA damage. Consequently, as shown in Figure 2A, DIC pretreatment significantly rescued MIA Paca‑2 cells from treatment A‑mediated cell death, confirming that the pulsed treatment is strictly NQO1‑dependent. However, DIC could barely inhibit treatment B‑induced cytotoxicity in both NQO1‑high MIA Paca‑2 cells and murine KPC cells (Figure 2A,B). Moreover, neither stable transfection with an NQO1 knockdown plasmid nor CRISPR‑mediated NQO1 knockout in MIA Paca‑2 cells could spare treatment B‑induced lethality (Figure 2C,D). Collectively, these results demonstrated that the cytotoxicity of the sustained low‑concentration β‑lap treatment did not rely on NQO1 biocatalysis. Instead, treatment B engaged a distinct, NQO1‑independent pathway that remained active even when NQO1 was genetically absent or pharmacologically inhibited. This unexpected finding prompted us to systematically identify the alternative molecular target responsible for the antitumor activity of this treatment.

**FIGURE 2:**
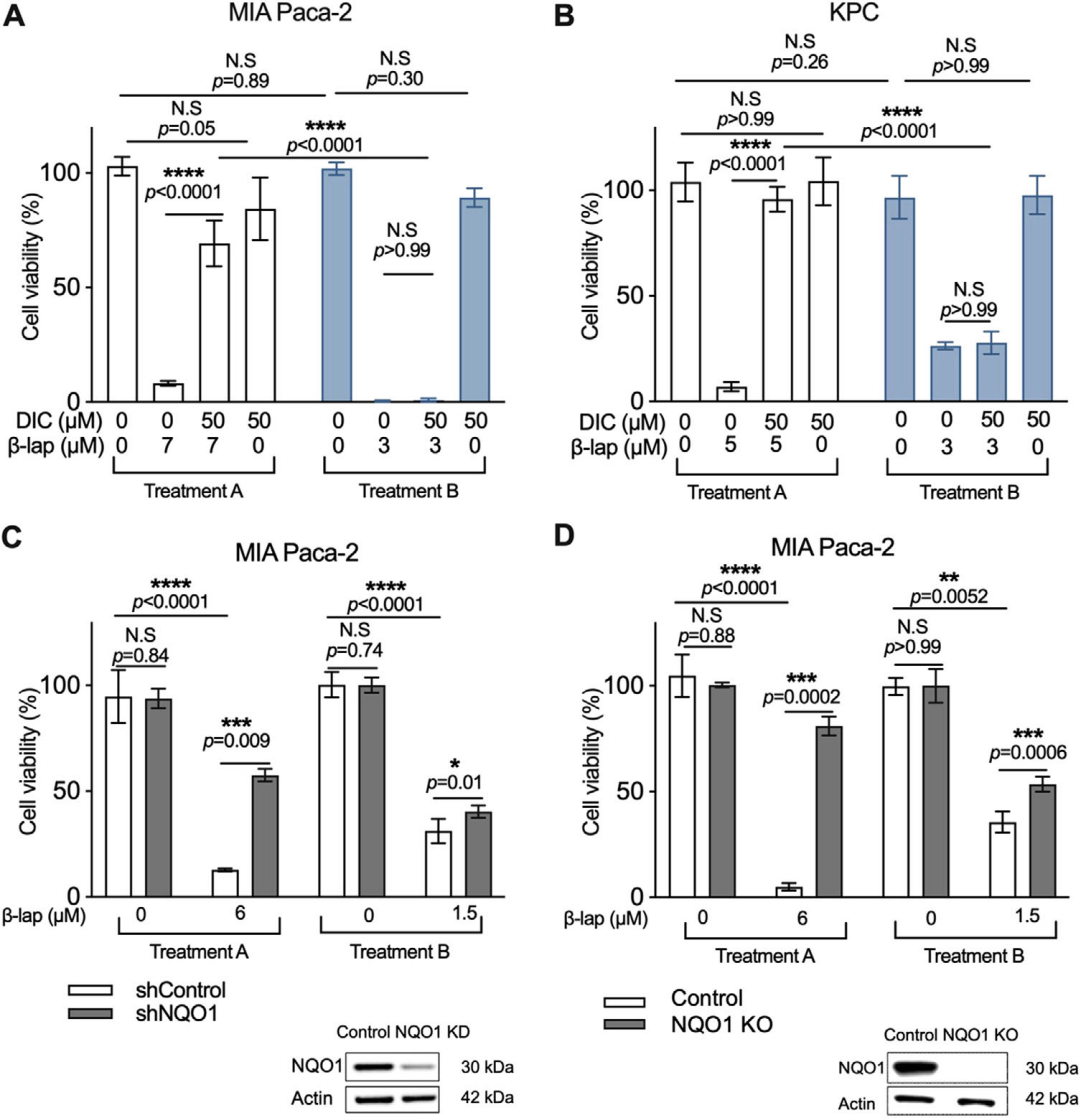
Sustained low‐concentration β‐lap induces cell death in an NQO1‐independent manner. (A) Survival assays of MIA Paca‐2 cells exposed to treatment A or treatment B of β‐lap with and without DIC (50 µM). (B) Survival assays of KPC cells exposed to treatment A or treatment B of β‐lap with and without DIC (50 µM). (C) Survival assays of MIA Paca‐2 cells with control or NQO1 knockdown shRNA exposed to treatment A or treatment B of β‐lap. (D) Survival assays of wildtype and NQO1 knockout MIA Paca‐2 cells exposed to treatment A or treatment B of β‐lap. *n* = 3, Data are expressed as mean ± SEM; ^*^ *p* < 0.05, ^**^ *p* < 0.01, ^***^ *p* < 0.001, ^****^ *p* < 0.0001 and N.S, not significant. Data were analyzed by two‐way ANOVA with Šídák's multiple‐comparisons test.

### 2.3 Vimentin Is Identified as a Direct Molecular Target of β‑Lap

To identify the target of β‐lap, we utilized a photoaffinity‐based pull‐down approach, in which covalent binding of β‐lap to target proteins was introduced via the photoactivatable crosslinker 3‐[3‐(but‐3‐yn‐1‐yl)‐3H‐diazirin‐3‐yl]propanoic acid. According to the results of a structure‐activity relationship study for β‐lap analogs [22, 23], a photoactive diazirine‐containing β‐lap probe (β‐lap‐P1) was designed and synthesized (Figure 3A and Figure S2A) with NMR verification (Figure S2B). β‐lap was not expected to form a stable covalent adduct with its targets, as confirmed by our MS analysis (Figure S8B). A diazirine was embedded as a photoreactive crosslinker, and an alkyne group was incorporated at the end as the moiety for the click reaction with azide‐linked fluorescence or biotin. After biotin conjugation, probe‐labeled proteins were enriched by streptavidin pulldown, allowing the enriched proteome to be identified by LC‐MS/MS. Notably, β‐lap‐P1 was also able to induce cell death in an NQO1‐independent manner (Figure S2C), suggesting that the introduction of the linker may not alter the binding target of β‐lap.

**FIGURE 3:**
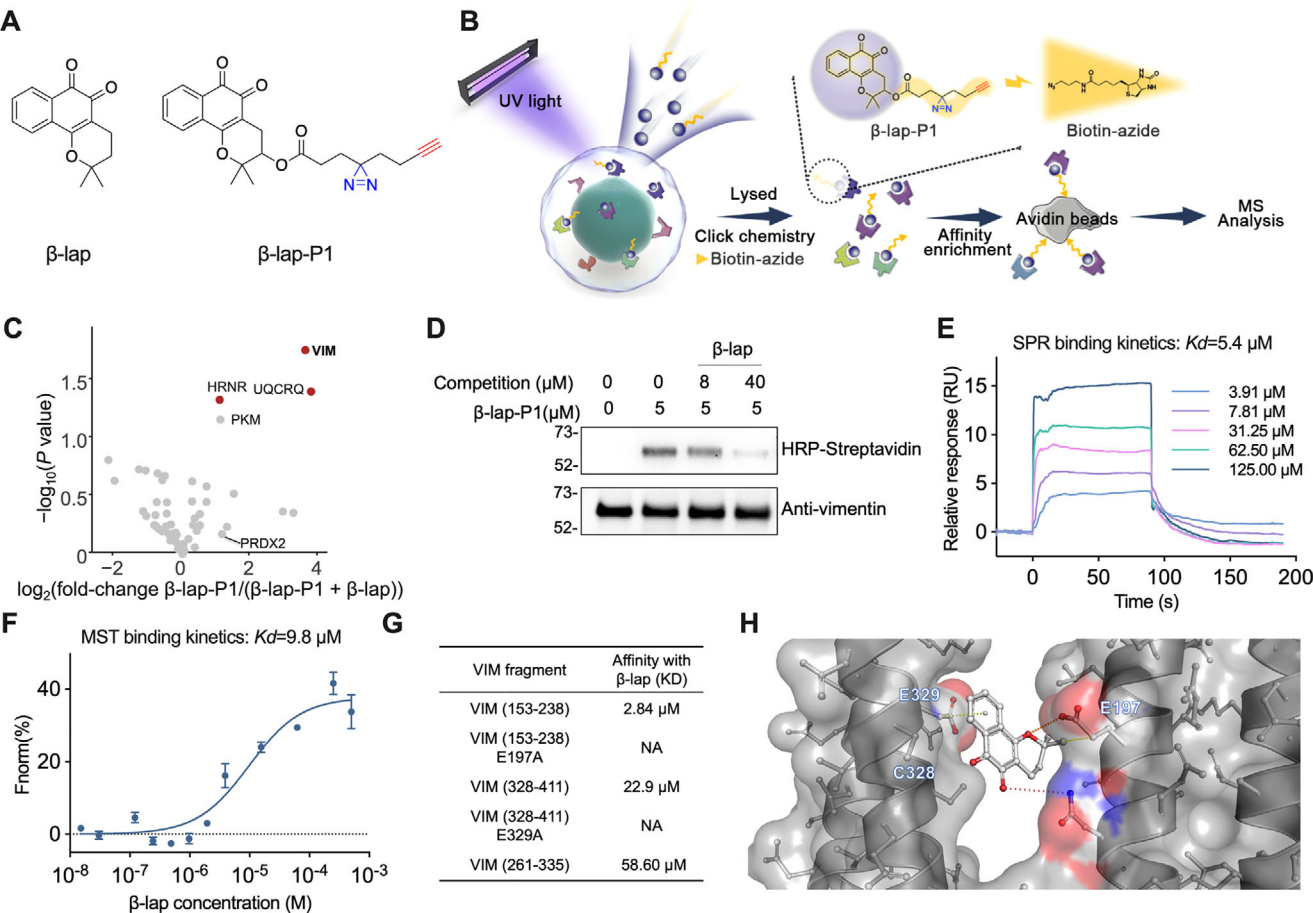
Identification and validation of vimentin as a direct target of β‐lap. (A) The chemical structures of β‐lap and β‐lap‐P1. The blue moiety represents a photoaffinity group. The red moiety is a tag for the click reaction. (B) A schematic diagram depicts the procedure of photoaffinity‐based pull‐down that used a photoactive β‐lap probe (β‐lap‐P1) from protein extracts of Panc1 cells. (C) A volcano plot of target identification by β‐lap‐P1 pulldown. The X‐axis displays the log_2_ fold change of proteins enriched by β‐lap‐P1 pulldown vs. pulldown in the presence of excess free β‐lap (competition). The Y‐axis shows ‐log_10_P. *n* = 3. (D) β‐lap‐P1‐labeled recombinant vimentin with or without excess β‐lap was western blotted with anti‐vimentin antibody or HRP‐streptavidin. (E) SPR results showing the relative response of vimentin incubated with β‐lap at indicated concentrations. (F) MST results of vimentin incubated with β‐lap at indicated concentrations for 3 h. (G) In SPR assays, VIM (328‐411), VIM (261‐335), VIM (153‐238), mutant VIM (328‐411) E329A, mutant VIM (153‐238) E197A were incubated with β‐lap at the indicated concentrations. The summarized affinity data are presented. (H) Computer modeling of the binding mode for the vimentin and β‐lap complex.

Given that sustained low‑concentration β‑lap treatment could kill tumor cells independent of NQO1, to explore the mechanisms beyond NQO1, we performed photoaffinity‐based pull‐down experiments in Panc1 cells. To exclude the nonspecific binding proteins, we employed parallel pull‐down experiments with or without β‐lap competition. After UV‐induced labeling of β‐lap‐P1 to target the proteome in Panc1 cells, cell lysates were biotinylated, and streptavidin beads were used to pulldown the β‐lap‐P1‐labeled proteomes for analysis by MS (Figure 3B,C and chemical reactions were shown in Figure S2D). The top proteins identified across three independent MS replicates were listed in Figure S2G, among which the binding of VIM was the most significantly competed by excess β‐lap and considered a potential target. Beyond VIM, only a few proteins showed reproducible enrichment that was competed by excess free β‐lap in all three replicate experiments. UQCRQ and HRNR fell into this category. However, UQCRQ is a structural subunit of mitochondrial complex III [24] and HRNR is a cornified‐envelope protein involved in epidermal barrier differentiation [25]. Little published evidence linked either protein to a functional target or β‐lap pharmacology. They were therefore not pursued. Moreover, it has been reported that PRDX1 knockdown potentiated β‐lap cytotoxicity by promoting ROS accumulation [26]. PRDX1 was not detected across three independent MS replicates, whereas PRDX2, a primary cytosolic H_2_O_2_ sensor [27], was competed by excess free β‐lap, albeit without statistical significance. However, PRDX2 knockdown did not alter cell viability under sustained β‐lap exposure (treatment B) (Figure S2H), whereas VIM depletion conferred marked resistance in Figure 5A. Affinity pull‐down assays further verified that β‐lap‐P1 could efficiently pulldown endogenous VIM from Panc1 cells (Figure S2E). We also observed that VIM was colocalized with fluorescence‐conjugated β‐lap‐P1 (Figure S2F).

To confirm the VIM binding of β‐lap directly, recombinant VIM was incubated with β‐lap‐P1 with or without β‐lap. The results showed that VIM efficiently bound with β‐lap‐P1 in a concentration‐dependent manner, and this binding was specifically competed by excess β‐lap, confirming direct interaction between β‐lap/β‐lap‐P1 and VIM (Figure 3D). Moreover, the binding affinity (*K_d_*) of β‑lap for vimentin was determined by surface plasmon resonance (SPR) and microscale thermophoresis (MST), yielding values of 5.4 µM and 9.8 µM, respectively (Figure 3E,F).

To map the β‑lap‑binding regions, three truncated VIM fragments were constructed, all retaining measurable binding affinity (Figure 3G and Figure S3A–D). Peptide mapping was then performed on recombinant VIM incubated with β‑lap‑P1, and the photocrosslinked β‑lap‑P1‐VIM complex was analyzed using LC‐MS/MS. A molecular weight increase of 379.15 Da was observed on two distinct peptides: 196‑EEAENTLQSFRQDVDNASLAR‑216 and peptide 322‑QVQSLTCEVDALK‑334. Within each peptide, the crosslink was mapped to one of several adjacent residues (E196/E197/E199 for the first peptide, and S325/T327 for the second), although the exact modification site could not be unambiguously assigned due to the inherent lack of residue specificity in photo‑crosslinking (Figure S8A). These data suggest that the β‑lap binding interface may reside within these two domains. On the basis of tagging sites, five spatially adjacent residues, E197, E329, K334, D367, and C328, were identified as potential binding sites for β‐lap‐VIM through molecular docking simulations. We generated five candidate residues for mutagenesis and observed that E197A or E329A mutations impaired the binding affinity of β‐lap to VIM, whereas mutations of other residues did not substantially reduce binding (Figure 3G and Figure S3E–J). These results indicate that β‐lap binds to VIM, with E197 and E329 serving as critical residues for the interaction. The β‑lap binding region on VIM was adjacent to C328 (Figure 3H), and the residue was subsequently established as the critical oxidation site. The carboxylate group of E329 may engage in an anion‐π interaction with the naphthoquinone aromatic ring, while E197 form a hydrogen bond with the pyran‐ring oxygen of β‐lap.

We next investigated whether β‑lap binding induces changes in VIM. Mass spectrometry analysis of tryptic peptides revealed oxidation modifications, manifested as characteristic +32/48 Da mass shifts, with no other types of chemical modifications detected (Figure S8B,C). The oxidation occurred in the absence of any enzymatic catalysts. The oxidized residues included the sole cysteine C328 and several methionine residues. C328 exhibited robust oxidation, whereas methionine oxidation was minimal and variable (Figure S3K). Although C328 lies adjacent to the β‐lap binding pocket (E197/E329), it does not participate in binding (Figure S3I). Instead, it serves as the primary oxidative modification site. This prompted us to investigate whether C328 oxidation of VIM triggered structural and functional consequences in cells.

### 2.4 β‑lap‐Induced Aggregation and Fragmentation of VIM Drive Ubiquitin‐Proteasome System Overload

C328, the sole cysteine of VIM, is essential for cytoskeleton organization and serves as a sensor for oxidative stress, with its selective modification known to disrupt filament assembly [28, 29, 30, 31]. We next examined whether sustained low‑concentration β‑lap treatment induced VIM structural and functional dysregulation in cells via C328 oxidation. Western blot results showed that exposure of Panc1 cells to β‐lap led to the formation of VIM oligomers of approximately 170 kDa under nonreducing conditions, as well as low molecular weight fragments of approximately 48 kDa, which showed an increase with higher concentrations and prolonged exposure time of β‐lap (Figure 4A,B and Figure S4A). The 170 kDa oligomers were compatible with a previously reported oxidized VIM dimer [32]. Owing to the outward orientation of cysteine residues in dimers, the 170 kDa band likely represented two covalently linked monomers originally belonging to adjacent dimers or tetramers that dissociated in SDS‐PAGE [29]. As expected, the oligomer band was undetectable under reducing conditions, where samples were pretreated with DTT to reduce disulfide bonds (Figure S4A), indicating that the 170 kDa oligomers were stabilized by intermolecular disulfide bonds.

**FIGURE 4:**
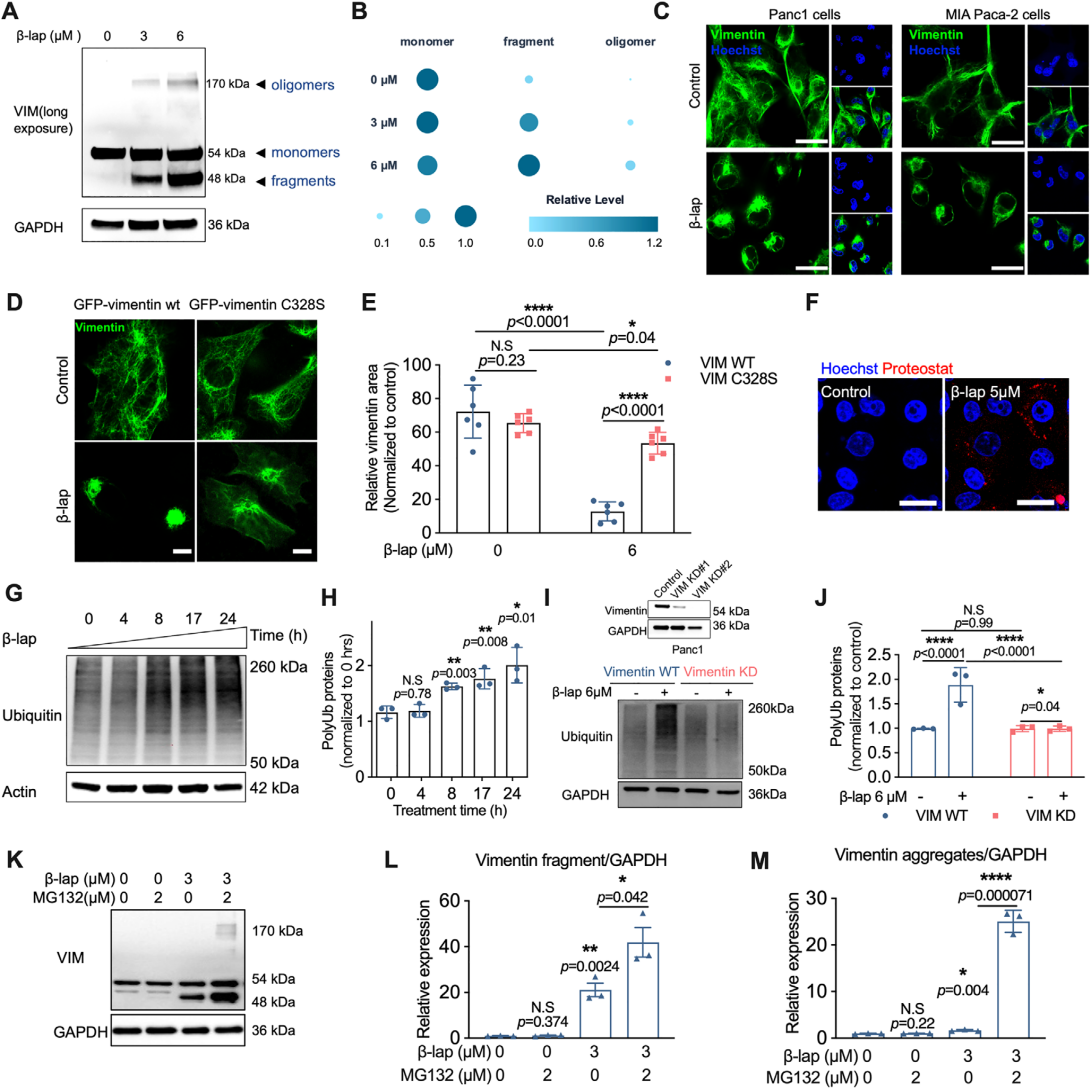
β‑lap‐induced aggregation and fragmentation of vimentin drive ubiquitin‐proteasome system overload. (A) Aggregation and cleavage of vimentin were increased in Panc1 cells after β‐lap treatment for 24 h in a concentration‐dependent way by western blotting. (B) Relative levels of vimentin oligomers, monomers, and fragments were quantified from (A) and are presented as a bubble plot, in which both the size and the color intensity of each bubble are proportional to the relative level. Levels were normalized to GAPDH and expressed relative to the monomer level of untreated (0 µM) cells (set to 1.0). (C) Confocal microscopy of vimentin (green) and Hoechst (blue) by immunofluorescence (IF) staining on Panc1 or MIA Paca‐2 after treatment with DMSO or β‐lap (treatment B) for 10 h. Representative single optical sections are shown. Scale bars, 20 µm. (D), Panc1 VIM knockdown cells transfected with wild‐type GFP‐vimentin or GFP‐C328S vimentin were treated with DMSO or β‐lap, and visualized by confocal microscopy. Scale bars, 20 µm. Quantification is shown in (E). Quantification was performed on six randomly selected fields per condition. *n* = 6. The levels of vimentin in this experiment are shown in Figure S4D. (F) Panc1 cells were treated with β‐lap for 5 h, stained with Hoechst (blue), proteostat (red). Scale bars, 20 µm. (G) Panc1 cells were treated with β‐lap for different durations and then analyzed for total‐fraction polyubiquitin levels via western blotting. Quantification is shown in (H). I, Wildtype Panc1 cells or vimentin knockdown Panc1 cells (KD#2) were treated with β‐lap and then analyzed for total‐fraction polyubiquitin levels via western blotting. Quantification is shown in (J). *n* = 3. The levels of vimentin in this experiment are shown in Figure S4E. (K) Protein levels of vimentin oligomer, monomers, and fragments were examined by western blotting after β‐lap with or without MG132 treatment for 20 h. Quantification is shown in (L–M). Results are representative of three independent experiments. All data are expressed as mean ± SEM; ^*^p < 0.05, ^**^p < 0.01, ^***^p < 0.001 and ^****^p < 0.0001; N.S, not significant. Data were analyzed by two‐tailed unpaired *t‐*test (H, L and M) and two‐way ANOVA with Šídák's multiple‐comparisons test (E and J).

Immunofluorescence showed that sustained low‐concentration treatment severely disrupted the VIM filament network, causing perinuclear condensation and cage‐like aggregates in both VIM‑high Panc1 and MIA Paca‑2 cells (Figure 4C). Withaferin A (WFA), a reported VIM inhibitor [33, 34], also showed similar structural changes (Figure S4B,C). To further evaluate the importance of C328 in β‑lap‑induced VIM dysregulation, we transfected GFP‑tagged VIM C328S mutant into VIM‑knockdown Panc1 cells. Notably, VIM C328S Panc1 cells were significantly resistant to β‑lap‑induced VIM aggregation and structural collapse (Figure 4D,E). Collectively, these data demonstrated that β‐lap‐provoked VIM network disruption relied primarily on oxidative crosslinking at C328, rather than on nonspecific cellular damage.

VIM has been reported to be a substrate of the ubiquitin‐proteasome system (UPS) and played a role in protein aggregates clearance. Upon β‐lap treatment, we observed a marked increase in protein aggregation (Proteostat staining) and total ubiquitinated protein levels, indicating UPS overload (Figure 4F–H). β‐lap (treatment B) triggered VIM aggregation and aggresome formation that colocalized with γ‐tubulin (Figure S4G). To determine whether this proteostatic stress was related to oxidative crosslinking and structural collapse of VIM, we knocked down VIM and found that it largely abrogated sustained low‐concentration treatment‐induced accumulation of ubiquitinated proteins, establishing aberrant reorganization of VIM filaments as the primary driver of proteostasis collapse (Figure 4I,J). To confirm whether residual VIM in knockdown cells mediated filament reorganization, we performed immunofluorescence, which revealed minimal residual VIM (Figure S4H) that remained unchanged upon β‐lap (treatment B). To further validate whether the proteasome is involved in the clearance of VIM aggregates and fragments, we pharmacologically blocked proteasome activity with MG132. Proteasome inhibition with MG132 further elevated VIM aggregates and fragments (Figure 4K–M) and synergistically enhanced cell death (Figure S5A). Consistently, the clinically approved proteasome inhibitors bortezomib and carfilzomib also synergized with β‐lap to enhance cytotoxicity (Figure S5B). Moreover, β‐lap‐induced increase in ubiquitinated proteins was significantly attenuated in C328S‐expressing cells compared with WT Panc1 cells (Figure S5C and D). This suggests that the β‐lap‐induced proteasome response is coupled to the oxidation and crosslinking of VIM at C328 and to the resulting aberrant VIM aggregation that drives proteostasis collapse. Thus, β‐lap‐induced VIM structural damage compromised proteasome capacity, and when this capacity was exceeded, it exacerbated cell death. To clarify the mechanism underlying VIM cleavage, we screened key protease families. Calpain, a calcium‐dependent cysteine protease known to cleave VIM [35, 36], was responsible, as its inhibitor MDL‐28170 reduced the fraction of VIM fragments relative to monomers (Figure S5E,F). In contrast, pan‐caspase inhibitor Z‐VAD‐FMK, lysosomal inhibitors (bafilomycin, 3‐MA) did not reverse cleavage (Figure S5G–L), excluding caspase or lysosome pathways. Meanwhile, cells initiated autophagy as a compensatory adaptive response to eliminate damaged proteins [37]. β‐lap (treatment B) also increased LC3‐II levels, and LC3 was recruited adjacent to vimentin aggregates, indicating the cells’ adaptive mechanism to clear the aggregated, ubiquitinated proteins to recover proteostasis (Figure S5M–O). Nonetheless, enhanced autophagic activity may fail to resolve overwhelming proteotoxic stress, as bafilomycin‐mediated lysosomal blockade showed limited impact on p62 turnover (Figure S5P,Q), indicating that the massive abnormal vimentin exceeded lysosomal degradative capacity.

### 2.5 VIM Is Essential for Sustained β‐Lap‐Mediated Cell Death

Given the findings that β‑lap binds to vimentin directly and sustained low‐concentration β‑lap leads to vimentin structural dysregulation, knockdown and overexpression experiments were performed subsequently to directly verify whether the cytotoxicity of this treatment is dependent on vimentin. We found that knockdown of VIM attenuated β‐lap (treatment B)‐induced cell death of Panc1 cells, as revealed by CCK8 assays and trypan blue staining (Figure 5A,B). Moreover, both KPC cells with CRISPR‑Cas9‑mediated VIM knockout and MIA Paca‑2 cells with shRNA‑mediated VIM knockdown exhibited resistance to β‑lap (treatment B) (Figure S6F,G). In addition, VIM knockdown in glioma U251MG cells increased the IC_50_ of β‐lap (treatment B) by more than ten‐fold, whereas VIM overexpression in VIM knockdown Panc1 cells restored β‐lap sensitivity (Figure 5C,D). Notably, this VIM‑dependent mechanism was operative only under the sustained low‑concentration treatment (treatment B), since VIM knockdown failed to protect cells from cell death induced by pulsed high‑concentration β‑lap (treatment A) (Figure S6H).

**FIGURE 5:**
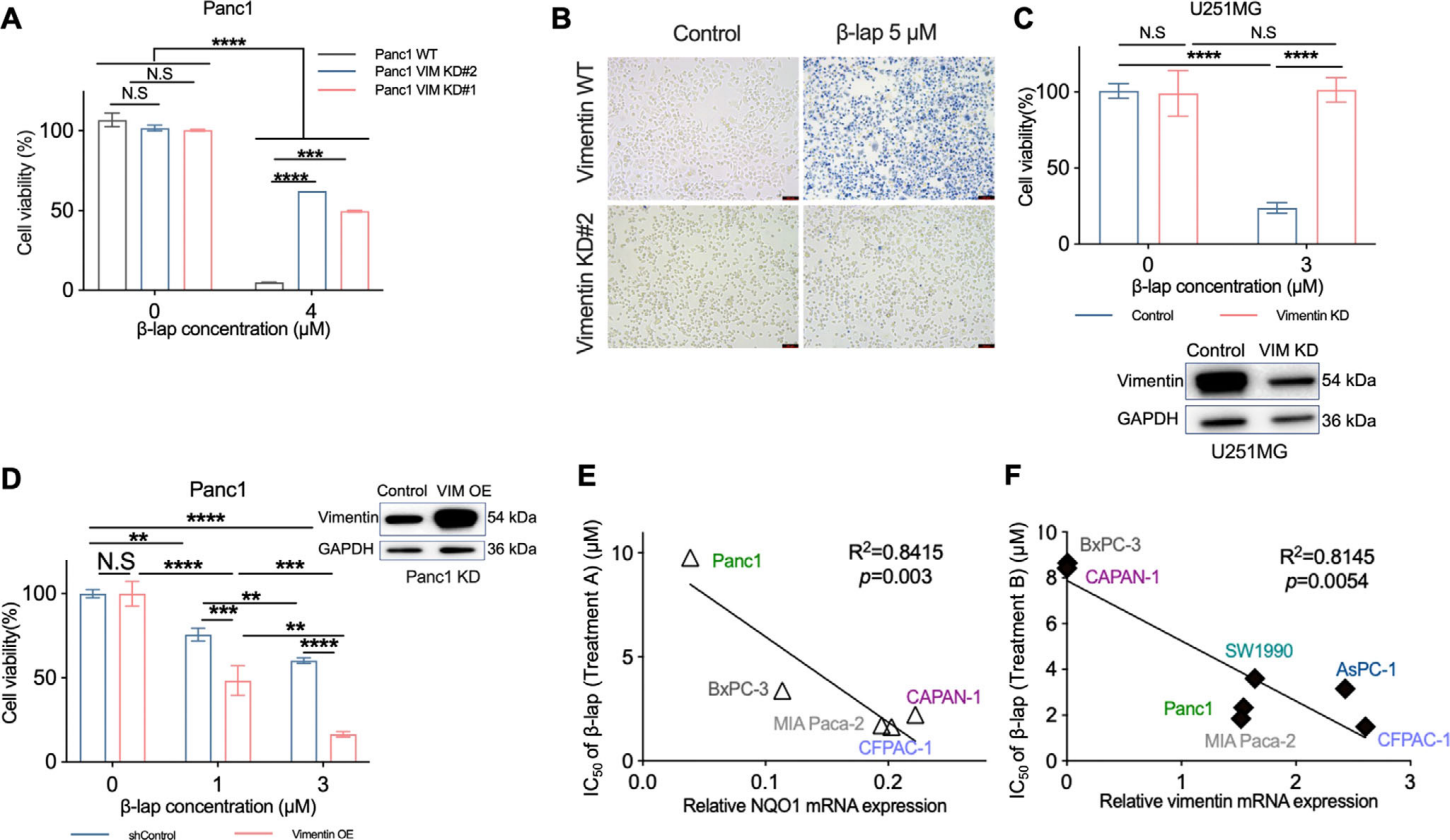
Vimentin is essential for sustained β‐lap‐mediated cell death. (A) Survival assays of VIM‐wildtype and VIM‐knockdown Panc1 clones exposed to β‐lap (treatment B). n = 3. (B) Trypan blue staining in wildtype and vimentin knockdown Panc1 cells treated with 5 µM β‐lap (treatment B). (C) Survival assays of wildtype and vimentin knockdown U251MG cells exposed to β‐lap (treatment B). *n* = 3. (D) Survival assays of VIM‐knockdown Panc1 cells (KD#2) transfected with a control plasmid or a plasmid overexpressing VIM, followed by exposure to β‐lap (treatment B). *n* = 6. (E,F) Correlation of sensitivity to β‑lap (treatment A) with NQO1 mRNA levels (E), and sensitivity to β‑lap (treatment B) with VIM mRNA levels (F), in pancreatic cancer cell lines. All data are expressed as mean ± SEM. ^*^ *p* < 0.05, ^**^ *p* < 0.01, ^***^ *p* < 0.001 and ^****^ *p* < 0.0001. Data were analyzed by two‐way ANOVA with Šídák's multiple‐comparisons test (A, C and D). Correlations in (E, F) were assessed by linear regression analysis between VIM/NQO1 mRNA levels and IC_50_.

In contrast, NQO1 knockout specifically protected cancer cells against pulsed high‐concentration β‑lap (treatment A) but failed to alter sustained low‐concentration β‑lap‐induced lethality (Figure 2D). These results demonstrated that the two treatment paradigms of β‐lap triggered independent downstream lethal cascades. Moreover, we evaluated endogenous VIM and NQO1 mRNA and their expression in a panel of human pancreatic cancer cells (Figure S6A,B). VIM‐high cell lines were markedly susceptible to β‐lap (treatment B), whereas VIM‐low cells displayed prominent resistance (Figure 5F). NQO1 expression correlated clearly with sensitivity to β‐lap (treatment A), consistent with previous reports (Figure 5E). Other reported β‐lap targets, including USP22 and topoisomerase I, showed no correlation with sensitivity to treatment B [38, 39] (Figure S6C).

To define the downstream death pathway, we performed unbiased transcriptomic analysis, which revealed distinct transcriptional signatures between the two groups: WT cells treated with β‐lap showed significant enrichment of pathways related to oxidative stress and ferroptosis. In contrast, signatures were quite different in Panc1 VIM knockdown cells, further confirming that VIM status globally rewrites the cellular response to β‑lap (Figure S6D). Moreover, ferroptosis was identified as a significantly enriched pathway (Figure S6I). Multiple ferroptosis‐associated gene sets were coordinately upregulated (Figure S6E), including glutathione metabolism genes (*SLC7A11, GCLC, GCLM, GPX3*), iron‐handling genes (*HMOX1, FTL, FTH1*), oxidative stress regulators (*TXNRD1, PRDX1*), and stress‐responsive transcription factors (*ATF4, JUN, ZEB1*). In addition, β‐lap (treatment B) markedly depleted intracellular GSH and dose‐dependently elevated MDA, a key marker of lipid peroxidation (Figure S6J,K). Functional validation using a panel of cell death inhibitors confirmed that ferrostatin‑1 efficiently rescued β‐lap (treatment B)‐induced cytotoxicity, whereas inhibitors of apoptosis, pyroptosis and autophagy showed little rescue effect (Figure S6L). This death pattern differed markedly from the conventional pulsed high‐concentration β‐lap treatment, which primarily drives programmed necrosis. Moreover, VIM knockdown substantially attenuated the upregulation of ferroptosis‑associated transcripts, including FTH1P7 and FTH1P11 (Figure S6M), suggesting that VIM‑driven UPS overload may serve as a proximal trigger for ferroptotic cell death.

### 2.6 Sustained Low‐Concentration β‐Lap Decreases Tumor Burdens in Mouse Tumor Models With High VIM Expression

Building on our findings in vitro that sustained low‐concentration β‐lap specifically targeted VIM to induce proteostasis collapse and cell death, we hypothesized that maintaining a low, stable concentration of β‐lap in vivo, similar to in vitro sustained low‐concentration β‐lap, could exert therapeutic tumor‐killing effects while reducing toxicity. To test this hypothesis, we designed an oral β‐lap formulation with a sustained‐release profile. Compared to intravenous injection of cyclodextrin‐formulated β‐lap (CD‐β‐lap), which exhibits a high C_max_ and rapid clearance, oral administration of sustained‐release β‐lap formulation achieved a flatter concentration‐time curve with lower C_max_ and prolonged half‐life (Figure 6A), recapitulating key features of the in vitro treatment B paradigm. In KPC mice, we detected strong VIM staining in primary tumors, liver metastases, and splenic metastases, but not in myocardium, liver parenchyma, or most renal cells (Figure S7A,B). Sustained low‑concentration β‑lap treatment significantly reduced tumor volumes and weights compared with the saline control (Figure 6C–E). Critically, the sustained low‐concentration β‐lap exhibited superior safety profiles. No significant differences were observed between the control and sustained low‐concentration β‐lap groups in body weight, organ index, serum ALT/AST levels, erythrocyte counts, or hemoglobin levels (Figure S7E–G), demonstrating low hepatorenal and hemolytic toxicity.

**FIGURE 6:**
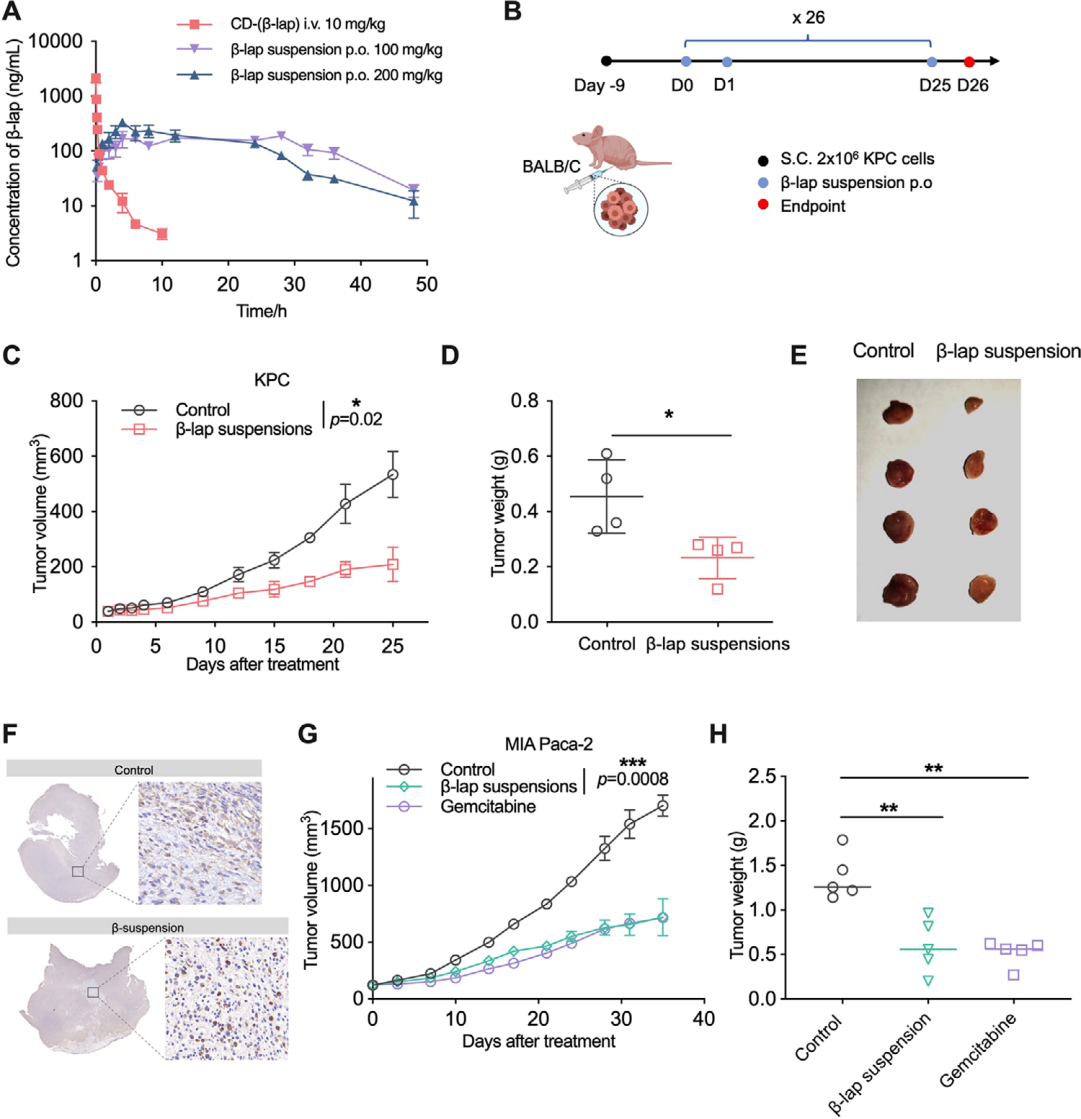
Sustained low‐concentration β‐lap decreases tumor burdens in mouse tumor models with high VIM expression. (A) Plasma concentration‐time curves of β‐lap following intravenous (i.v.) administration of CD‐β‐lap at 10 mg/kg or oral (p.o.) administration of β‐lap at 100 and 200 mg/kg. Data are presented as mean ± SEM. *n* = 3. (B) Schematic of the in vivo antitumor efficacy study. BALB/c nude mice were subcutaneously (s.c.) inoculated with 2 × 10^6^ KPC cells on day ‐9, followed by daily oral (p.o.) administration of sustained‐release β‐lap formulation from day 0 to day 25 (×26 doses). The endpoint was designated on day 26. (C–E) KPC tumor‐bearing mice were administered saline or sustained β‐lap 100 mg/kg once a day. Tumor growth curves (C) and tumor weight changes (D), photographs of excised tumors at the endpoint (E) of mice are shown. (*n* = 4 per group). (F) Representative IHC staining of vimentin in tumor tissues in KPC tumor‐bearing mice treated with saline or sustained β‐lap 100 mg/kg. (G) BALB/c nude mice were subcutaneously inoculated with 1 × 10^6^ MIA Paca‐2 cells, and tumor‐bearing mice were administered saline, sustained β‐lap 100 mg/kg twice a day, or gemcitabine 50 mg/kg intravenously twice a week for 4 weeks, and tumor growth curves were shown. (H) Tumor weight changes of the mice treated with sustained β‐lap or gemcitabine in mouse models in (G). (*n* = 5 mice per group in G–H.) All data are presented as mean ± SEM; ^*^ *p* < 0.05, ^**^ *p* < 0.01, ^***^ *p* < 0.001 and ^****^ *p* < 0.0001; N.S, not significant. Endpoint of tumor growth curves in (C and G) and tumor weights in (D and H) were analyzed by the Mann‐Whitney *U*‐test.

To study the involvement of VIM in the mechanism of action (MoA) of sustained β‑lap in vivo, we established xenografts from VIM‐low NCI‐N87 and VIM‐high A549 cells. When treated with the sustained low‐concentration β‐lap, NCI‐N87 xenografts showed no significant tumor growth inhibition compared to controls (Figure S7I,J), but the first‐line agent paclitaxel significantly inhibited tumor growth. By contrast, lung cancer A549 xenografts, which express high VIM levels, displayed robust antitumor responses (Figure S7K,L). Notably, NQO1 mRNA levels were comparable among MIA Paca‐2, NCI‐N87, and A549 cells according to the Human Protein Atlas database. These results suggest that VIM expression, rather than NQO1 status, may be the primary determinant of in vivo sensitivity to the sustained low‐concentration treatment. Given that the two cell lines differ in many aspects besides VIM expression levels, to confirm that the in vivo anti‐tumor effects were mediated by VIM targeting, we analyzed tumor tissues post‐treatment. After sustained low‐concentration β‐lap administration, VIM was also decreased in KPC tumors and showed condensation into punctate aggregates, further validating the VIM targeting mechanism in vivo (Figure 6F). Accordingly, the increase in the 20S α5 proteasome subunit post‐treatment suggested compensatory upregulation of proteasome capacity, potentially facilitating the clearance of β‐lap‐induced aggregates and fragments (Figure S7M). This aligned with the observed VIM condensation and supported proteasome‐dependent degradation as a mechanism of action. VIM overexpression has been reported to promote epithelial cell migration, invasion, metastasis, and stemness. Notably, treatment with β‐lap also upregulated E‐cadherin expression (Figure S7M), suggesting a potential reversal of EMT. Furthermore, in MIA Paca‐2 xenograft models, sustained low‐concentration β‐lap administration also exhibited potent tumor growth inhibition, comparable to gemcitabine, the first‐line clinical therapy for pancreatic cancer (Figure 6G,H and Figure S7H). Collectively, oral administration of the rationally designed β‑lap formulation achieved sustained low‑concentration PK profiles in vivo and exhibited anti‑tumor effects with improved safety in xenograft models of pancreatic cancers with high vimentin expression.

## 3 Discussions

The results presented here fundamentally reframe our understanding of β‐lap as an antitumor agent. For decades, the biological activity of β‐lap has been attributed almost exclusively to its role as a substrate for NQO1‐mediated redox cycling, which generates cytotoxic ROS bursts sufficient to induce programmed necrosis in NQO1 overexpressing tumors. Although this mechanism is well‐validated, it carries two intrinsic limitations. First, since cytotoxicity is contingent on NQO1 bioactivation, tumors with low or absent NQO1 expression are inherently refractory to treatment, confining the clinical utility of β‐lap. Second, achieving therapeutically effective ROS bursts requires pulsed high‐concentration administration, which inevitably generates off‐target oxidative stress in erythrocytes, causing dose‐limiting hemolytic anemia and methemoglobinemia that severely narrow the therapeutic window. Here, our findings reveal that both limitations can be circumvented, not by structural modification of the compound, but by reprogramming its pharmacokinetic profile. The sustained treatment not only redirects the mechanism of action from NQO1‐ROS to VIM‐driven proteostasis collapse, but also expands the therapeutic window by enabling efficacy at much lower, subtoxic concentrations. This dual benefit, mechanistic switching and safety expansion, provides new insight for indication and patient selection during clinical translation of β‐lap.

C328 is rendered unusually nucleophilic by the electrostatic environment of the coiled‐coil. Electron transfer from the C328 thiolate to β‐lap initiates a local redox cycle that generates peroxide, oxidizing C328 to a sulfenic acid (–SOH) intermediate [40, 41, 42] that condenses with the C328 thiolate of a neighboring vimentin monomer to form a reversible intermolecular disulfide (Cys‐S‐S‐Cys’). The sensitivity to DTT indicates that the oligomers are stabilized by reversible disulfide linkages, consistent with the oxidation of C328.

Reducing drug exposure is a straightforward strategy to improve safety for drugs with a narrow therapeutic index. However, when both efficacy and toxicity depend on high‐concentration pulses, lowering C_max_ risks efficacy loss. For β‐lap, numerous formulation efforts (prodrugs, micelles, nanoparticles) have aimed to expand the therapeutic window, but they generally attributed improvements to altered biodistribution or reduced systemic toxicity rather than considering potential changes in the drug's mechanism of action. Most delivery studies have implicitly assumed that the NQO1‑dependent mechanism remains the sole operative pathway. Our findings establish a new paradigm by revealing that modulating the pharmacokinetic exposure profile does not merely reduce C_max_, but actively redirects β‐lap's primary target engagement from a narrowly applicable NQO1‐dependent ROS producer to a broadly effective, VIM‐targeted oxidant. While dose‑dependent mechanism switching is a well‑established pharmacological principle (e.g., aspirin, dopamine [43], bupropion [44], quetiapine [45], metformin [18]), our work provides a paradigm for intentionally leveraging this principle to expand the therapeutic window of a single agent through rational pharmacokinetic design.

The identification of VIM as a direct molecular target of β‐lap under sustained low‐concentration conditions is a significant and unexpected finding. VIM is a core cytoskeletal regulatory protein, which maintains cellular morphology, division and organelle spatial distribution [46, 47, 48, 49, 50]. Mounting evidence has established its multifaceted contributions to cancer progression, including tumor invasion, metastasis and therapeutic resistance [51, 52, 53, 54, 55]. Despite its potential as a therapeutic target [56, 57, 58], VIM has remained challenging to target due to its lack of conventional binding pockets and conformational plasticity [32]. The present study, leveraging photoaffinity labeling with β‐lap‐P1 followed by mass spectrometry‐based proteomics, provides direct biochemical evidence that β‐lap engages VIM with micromolar affinity, as corroborated by orthogonal biophysical methods including SPR and MST. Time‐lapse imaging revealed a progressive process: perinuclear aggregates appeared at 5 h, fragmented by 15 h, and intensified with cell shrinkage at 25 h (Figure S4F). Human tumor tissues also showed elevated VIM expression compared with normal counterparts (Figure S7C), and high VIM levels correlated with poor prognosis in pancreatic cancer and glioma (Figure S7D), validating VIM as a potential therapeutic target. In our experiments, KPC cell‐derived xenograft with stable VIM knockout markedly attenuated tumor growth in vivo (data not shown), consistent with the recognized role of vimentin in sustaining the malignant phenotype. Thus, the residual anti‐cancer activity of β‐lap in VIM‐deficient KPC tumor would be difficult to resolve against the strongly impaired baseline tumorigenicity. Given that the CDX models used in this study differ in multiple genetic and phenotypic features besides VIM expression, the present data may nevertheless provide indirect evidence that VIM expression, rather than NQO1 status, is the primary determinant of in vivo sensitivity to the sustained low‐concentration treatment.

The PK‐dependent mechanism switching observed here is governed by the interplay between target abundance and pharmacokinetic characteristics. In cells expressing NQO1 only (e.g., CFPAC‐1), pulsed high concentration drives ROS‐mediated death, whereas sustained low concentration is ineffective (Figure 5). In cells expressing vimentin only (e.g., Panc1), sustained low‐concentration kills via gradual vimentin crosslinking accumulation. The affinity of β‐lap for VIM is in the micromolar range (Figure 3E,F), which is comparable to its affinity for NQO1 (data not shown). The in vitro binding assays were performed with an N‐terminally his‐tagged construct, and we note that tag‐mediated perturbation of the in vitro assembly and binding behavior cannot be excluded. Nonetheless, this tagged VIM retained the ability to form intermediate filaments. It is therefore plausible that, in cells expressing both VIM and NQO1, β‐lap can interact with both targets. The pharmacological effects arising from these two mechanisms, NQO1‐dependent ROS production and VIM oxidation, may operate concurrently, with the PK profile determining their relative contribution. At high peak concentrations (C_max_‐driven), NQO1 is kinetically saturated, driving rapid and massive ROS production. At lower, sustained concentrations, ROS generation is insufficient to trigger the necrotic cascade, yet β‐lap accumulates to levels sufficient for productive crosslinking of VIM, which is a slower, stoichiometry‐dependent process. This mechanistic bifurcation is consistent with the distinct dose‐response profiles observed in NQO1‐high vs. VIM‐high cell lines and underscores the necessity of defining PK characteristics as part of the mechanistic characterization of multi‐target compounds. These findings also suggest a broader principle: rapid‐acting targets (e.g., kinases, DNA damage, acute ROS) respond to transient high‐concentration exposure, whereas slow cumulative processes (e.g., proteostasis collapse and redox exhaustion) require sustained intervention. Future studies employing pharmacokinetic/pharmacodynamic (PK/PD) modeling will be essential to precisely delineate these boundaries and guide clinical translation. Furthermore, the broad overexpression of VIM in EMT‐driven cancers suggests that the sustained‐release formulation of β‐lap may be particularly advantageous in the setting of acquired drug resistance, where EMT‐associated VIM upregulation is a common adaptive response to conventional chemotherapy and targeted agents.

Despite the absence of a classical ligand‐binding pocket [59], β‐lap could engage tetrameric VIM through an interface in our docking model. This is consistent with previous reports showing that Withaferin A, a known vimentin inhibitor [33, 34], is proposed to covalently bind to tetrameric vimentin, and that the fluorescent chemical probe TiY exhibits higher affinity for tetrameric vimentin than for its monomeric counterpart [60]. In addition, we propose that VIM‐associated proteins may also create a transient pocket or guide the interaction. Given that VIM often functions within heteropolymeric networks in different cellular contexts, notably, U251MG cells also express GFAP, another type III intermediate filament, which can heterooligomerize with vimentin. The attenuated β‐lap toxicity upon VIM knockdown might be partly related to the disruption of the VIM‐GFAP interaction, which warrants further investigation.

This PK‐associated MoA shift of β‐lap is not an isolated case. For example, low‐dose metformin promotes longevity through a PEN2‐dependent pathway rather than its conventional AMPK activation [18]. Aspirin exerts anti‐inflammatory effects at low doses and anti‐platelet effects at higher doses [61, 62]. Notably, even monoclonal antibodies with high target specificity can elicit complex pharmacological responses due to ligand‐receptor promiscuity and signaling crosstalk. For example, ustekinumab, an antibody targeting the p40 subunit, blocks both IL‐12 and IL‐23 signaling because p40 is a shared subunit of both cytokines, a feature that confers dual pathway modulation beyond its intended single‐target design [63, 64]. Furthermore, the pharmaceutical landscape is rapidly evolving, with innovations in formulations and administration routes reshaping clinical practice, as exemplified by subcutaneous atezolizumab [65, 66] and oral semaglutide [67]. Thus, formulation innovations do not merely alter bioavailability but also reshape PK profiles, underscoring that mechanism of action and target preference are dynamic attributes profoundly influenced by dosing schedule, formulation, and route of administration.

Although our data establish VIM as a key direct target mediating sustained low‐concentration β‐lap cytotoxicity, other cellular proteins or pathways may also contribute. For instance, while VIM knockdown largely abrogated sustained low‐concentration β‐lap lethality, the protective effect was incomplete at higher drug concentrations, suggesting the involvement of additional targets or off‐target effects. Future studies using unbiased proteome‑wide oxidative modification profiling may reveal other cysteine‑sensitive proteins that cooperate with VIM to execute ferroptosis. Nonetheless, these considerations do not diminish the central conclusion that PK‑driven VIM targeting represents a viable and novel therapeutic strategy for VIM‑high cancers.

## 4 Conclusions

In summary, this study establishes that pharmacokinetic tuning is a determinant of mechanism selectivity for the antitumor natural product β‐lap. By maintaining sustained low plasma levels with an optimized release formulation, β‐lap bypasses its canonical NQO1‐dependent mechanism and instead engages VIM as its primary target. β‐lap acts as a direct oxidant of VIM, oxidizing C328 directly and inducing VIM aggregation, collapsing cellular proteostasis, and ultimately triggering ferroptotic cell death. This VIM‐targeting axis remains fully functional in NQO1‐deficient cancer cells. Translational validation in MIA Paca‐2 pancreatic cancer xenograft models demonstrates that the sustained‐release formulation achieves antitumor efficacy comparable to gemcitabine at its maximum tolerated dose, with a favorable organ safety profile. These results provide a compelling preclinical rationale for the clinical development of PK‐optimized β‐lap in VIM‐high malignancies, including PDAC, TNBC, and glioma.

Beyond β‐lap itself, this work articulates a broader principle: the PK profile of a bioactive molecule is an underexplored yet programmable dimension of its pharmacology. As demonstrated here, PK re‐engineering can unmask latent mechanisms, broaden the target landscape, and improve the therapeutic index of existing compounds without structural modification. We propose that systematic evaluation of natural products and clinical drugs across diverse PK profiles represents a productive and largely unexplored strategy for drug repurposing and mechanism discovery. This paradigm may prove especially valuable for the large class of multi‐target compounds whose full therapeutic potential remains incompletely characterized.

## 5 Materials and Methods

Supplier and catalog number information for all key reagents (antibodies, chemicals, peptides, and recombinant proteins, critical commercial assays, and recombinant DNA related to Methods) throughout the manuscript is listed in Table S4. Primers used for RT‐qPCR analysis and siRNA/shRNA/gRNA sequences are listed in Tables S1–S3.

### 5.1 Cell Culture

The BxPC‐3 (ATCC, Cat# CRL‐1687, RRID:CVCL_0186) and AsPC‐1 (Procell Life Science & Technology, Cat# CL‐0027, RRID:CVCL_0152) cell lines were grown in RPMI 1640 medium (Gibco, Waltham, USA). MIA Paca‐2 (ATCC, Cat# CRL‐1420, RRID:CVCL_0428), CAPAN‐1 (ATCC, Cat# HTB‐79, RRID:CVCL_0237), A549 (ATCC, Cat# CCL‐185, RRID:CVCL_0023), NCI‐N87 (ATCC, Cat# CRL‐5821, RRID:CVCL_1603), CFPAC‐1 (ATCC, CRL‐1918, RRID:CVCL_1116), SW1990 (ATCC, Cat# CRL‐2172, RRID:CVCL_1723), U251MG (Procell Life Science & Technology, Cat# CL‐0237, RRID:CVCL_0021) and Panc1 (ATCC, Cat# CRL‐1469, RRID:CVCL_0480) cell lines were cultured in high glucose Dulbecco's modified Eagle's medium (DMEM, Gibco, Waltham, USA). Murine KPC cell lines were originally derived from tumors of a genetically engineered mouse model of pancreatic cancer, *LSL‐Kras^G12D/^* ^+^ *; LSL‐Trp53^R172H/^* ^+^ *; Pdx‐1‐Cre* (KPC) mice, and were maintained in DMEM. All media were supplemented with 10% FBS and 1% penicillin–streptomycin (Gibco). All cell lines were authenticated by short tandem repeat (STR) profiling.

### 5.2 Animal Models

Male Sprague Dawley rats (200‐250 g) were used for pharmacokinetic studies. KPC mice (*LSL‐Kras^G12D/^* ^+^ *, LSL‐Trp53^R172H/^* ^+^ *, Pdx‐1‐Cre*) were generated by crossing *LSL‐Kras^G12D/^* ^+^ *, LSL‐Trp53^R172H/^* ^+^ mice and *Pdx‐1‐Cre* mice, genotyped by PCR analysis of tail genomic DNA as previously described [68]. After spontaneous pancreatic tumors developed, tumor‐bearing KPC mice (12–16 weeks of age, 20–30 g, both male and female) were euthanized, and pancreatic tumors were excised and enzymatically digested to isolate KPC‐derived tumor cells, following previously established procedures [69]. KPC tumor cells, MIA Paca‐2, NCI‐N87, A549 tumor cells were subcutaneously implanted into female BALB/c nude mice (6 weeks old, 18–22 g), and the mice were randomized into the indicated treatment groups once the tumor volume reached 50–100 mm^3^. All animals were housed under specific pathogen‐free conditions with a 12‐h light/dark cycle and free access to food and water. All experimental procedures were conducted in accordance with the Institutional Animal Care and Use Committee (IACUC) guidelines and approved by the Animal Ethics Committee of Tsinghua University under Animal Protocol Number 16‑QF1 (Title: *In vivo evaluation of anti‑cancer drug delivery system in lung cancer, PDAC and colon cancer*).

### 5.3 Method Details

#### 5.3.1 Knockdown and Knockout Experiment

Lentiviral shRNA‑mediated knockdown. Lentiviral particles packaged in HEK293T cells (pLKO.1‐puro‐NQO1‐shRNA plus the packaging plasmids psPAX2 and pMD2.G) were used to transduce MIA Paca‐2 cells as previously described [13]; stable knockdown pools were selected with puromycin (1 µg/mL). Scrambled shRNA served as the non‐targeting control.

CRISPR‑Cas9‑mediated knockout. NQO1 or VIM knockout was generated by transfecting MIA Paca‑2 cells with Cas9 and gene‑specific sgRNA plasmids. Single‑cell clones were isolated by FACS, and knockout was confirmed by Western blotting. Non‑targeting sgRNA was used as a control.

Transient knockdown/overexpression. Plasmids were delivered using Lipofectamine 3000 (Invitrogen) per the manufacturer's protocol. Cells were harvested at 72 h (knockdown) or 48 h (overexpression) post‑transfection.

### 5.4 Trypan Blue Staining

Trypan blue staining was performed to assess cell viability. Cells were incubated in 12‐well plates at 2.0 × 10^5^ cells per well and treated with β‐lap (treatment B). Adherent cells were stained with 0.4% trypan blue (VWR, China) at room temperature for 10 min and observed under a microscope (TE2000‐S; Nikon, Tokyo, Japan). Live cells, which possessed intact cell membranes, excluded trypan blue and remained unstained, while dead cells with compromised membranes took up the dye and were stained blue.

### 5.5 Cellular ROS Detection

Cellular ROS was determined using the DCFDA‐Cellular ROS Detection Assay Kit (Abcam) according to the manufacturer's instructions. Briefly, adherent Panc1/MIA Paca‐2 cells were plated into a dark, clear bottom 96‐well microplate with about 70% confluence (4000 cells/well) and adhered overnight. After the culture media was removed, cells were stained with diluted DCFDA solution for 45 min at 37°C in the dark and treated with β‐lap or H_2_O_2_ for different durations. ROS signal of the plate was measured immediately on a fluorescence plate reader at E_x_/E_m_ = 485/535 nm.

### 5.6 Synthesis of the β‐lap‐P1

The photocrosslinker probe was prepared following previously established procedures with slight modifications [22, 23]. Lapachol (30.2 mg, 1.0 eq) was dissolved in CH_2_Cl_2_ (3 mL), and then meta‐Chloroperoxybenzoic acid (m‐CPBA, 25.8 mg, 1.2 eq) was added. The reaction mixture was stirred at room temperature (RT) for 30 min. Then, trifluoroacetic acid (TFA, 29 µL, 3.0 eq) was added, and stirring was continued for 1 h. The resulting solution was poured into distilled water and extracted three times with CH_2_Cl_2_. The combined organic phases were washed sequentially with saturated aqueous NaHCO_3_ solution (three times) and brine (once), dried over anhydrous MgSO_4_, filtered, and concentrated under reduced pressure. The crude residue was purified by silica gel column chromatography eluting with petroleum ether/ethyl acetate (PE/EA = 1:1, v/v) to afford Stenocarpoquinone A as a red solid (18.7 mg, 58.7% yield).

3‐[3‐(but‐3‐yn‐1‐yl)‐3H‐diazirin‐3‐yl]propanoic acid (24.2 mg, 2.0 eq) was dissolved in CH_2_Cl_2_ (6 mL), and then EDCI (41.6 mg, 3.0 eq), DMAP (26.5 mg, 3.0 eq) were added. The mixture was stirred at RT for 1 h. Stenocarpoquinone A (18.7 mg, 1 eq) was then added, and the reaction was stirred at RT for an additional 4 h in the dark (to prevent photodegradation of the diazirine moiety). The reaction mixture was dried over MgSO_4_, filtered and concentrated under reduced pressure. The crude residue was purified by silica gel column chromatography eluting with PE/EA = 2:1 (v/v) to yield the target product as an orange solid (16.2 mg, 55.1% yield).

### 5.7 Cell Viability Assay

Cell viability was measured by a CCK‐8 kit (Beyotime Biotechnology). Cells were seeded into 96‐well plates (6000 cells per well in 0.1 mL medium) and, after overnight attachment, exposed to the indicated compounds; CCK‐8 reagent was then added to the wells for 2 h, and absorbance at 450 nm was recorded. Each sample condition was set up in at least three replicate wells, and the experiment was repeated three times. The dose–response curves and IC_50_ values were derived using GraphPad Prism software with the non‐linear regression “(inhibitor) vs. normalized response (variable slope)” protocol.

### 5.8 β‐Lap Treatment Schedules

For treatment A, cells were exposed to β‑lap for 5 h, after which the drug‑containing medium was removed and replaced with fresh drug‑free medium for the remaining 20 h. For treatment B, cells were incubated with β‑lap at a lower concentration for 9 h, after which a small volume of concentrated β‑lap stock solution was added to the culture medium to replenish the drug level and maintain the intended lower concentration for another 9 h; this replenishment cycle was repeated once more at 18 h to achieve a total of three exposures within 24 h.

### 5.9 Western Blotting

For immunoblotting, cells were lysed in RIPA buffer supplemented with a protease inhibitor cocktail. After protein concentrations were normalized, each was mixed with 5 × SDS loading buffer (with DTT, Yeasen) and denatured at 95°C for 5 min under reducing conditions. Protein lysates were mixed with 5 × SDS nonreducing loading buffer without DTT at 90°C for 2 min under nonreducing conditions. Then protein samples were resolved on an SDS–PAGE gel (GenScript). Gels were transferred to activated 0.45 µm PVDF membranes (Immobilon‐P) using a wet transfer system (Bio‐Rad). The membranes were blocked for 1 h at room temperature with 5% (w/v) milk in Tris‐buffered saline (TBS) while rocking. Primary antibodies were applied in Antibody Diluent (Solarbio) overnight at 4°C, followed by five 5‐min washes with TBST (TBS with 0.1% Tween‐20). Secondary antibodies were then applied in Antibody Diluent (Solarbio) for 2 h at room temperature, followed by five 5‐min washes with TBST. The membrane was imaged using an Imaging System. Image J was used to normalize and quantify lane intensities.

### 5.10 β‐Lap‐Binding Proteins Identification

The 15‐cm dishes of Panc1 were treated with DMSO, β‐lap‐P1 with or without excess β‐lap for 4 h, and then exposed to 365‐nm wavelength UV (CX‐2000, UVP) for 20 min. Twenty 15‐cm dishes of Panc1 cells of each treatment condition were lysed with ice‐cold HEPES lysis buffer (50 mM HEPES, pH 8.0, 1.5 mM MgCl_2_, 10 mM KCl, 1% SDS with protease inhibitor cocktail and 0.1 U/µL Benzonase) and rotated for 1 h at room temperature. After centrifugation, supernatants were diluted into about 1 mg/ml and incubated with 1 mM TCEP, 102 µM TBTA, 1 mM CuSO_4,_ and 100 µM biotin‐N_3_ at room temperature for 1 h with rotation. Acetone was added to the samples, and protein aggregates were separated by centrifugation at 8000 g for 15 min. Streptavidin beads (Dynabeads M‐280) were then added to the protein samples and incubated overnight with gentle rotation. The beads were washed 3 times with 7 M Urea, 4% SDS buffer, then mixed with 5 × SDS loading buffer (Yeasen, containing DTT) before SDS gel electrophoresis. The gels were stained for 30 min with a solution of Coomassie Brilliant Blue R‐250 and decolorized using water, followed by MS analysis. Excised gel lanes were destained with 50% acetonitrile/50 mM ammonium bicarbonate, reduced with 5 mM DTT at 45°C for 30 min, and alkylated with 11 mM iodoacetamide. In‐gel digestion was performed using trypsin (Promega) at 37°C overnight. Peptides were extracted twice with 50% acetonitrile / 0.1% formic acid, dried under vacuum. LC‐MS/MS analysis was performed on an Orbitrap Fusion Lumos Tribrid mass spectrometer (Thermo Fisher Scientific) coupled to a nanoLC system. Peptides were separated on a C18 column using a gradient of 80% acetonitrile in 0.1% formic acid and analyzed in data‐dependent acquisition mode.

Raw MS data were processed using Proteome Discoverer against the UniProtKB/Swiss‐Prot database for Homo sapiens. Peptide and protein identifications were validated at a FDR < 1% using a target‐decoy approach. Proteins were accepted if they met the following criteria: at least one peptide and one unique peptide, and a Score Sequest > 5.

### 5.11 Co‐Immunoprecipitation and Immunoblotting

For validation of β‐lap binding by pull‐down, recombinant vimentin was incubated with DMSO (vehicle) or with 5 µM β‐lap‐P1 together with 8 µM or 40 µM β‐lap or 10 µM WFA competition for 2 h. After UV‐induced labeling, proteins were conjugated with biotin and purified over streptavidin beads. Then they were subjected to SDS‐PAGE and western blotted with anti‐vimentin antibody or HRP‐streptavidin.

For in‐cell validation, Panc1 cells (grown to 60% confluence) were transfected with vimentin overexpression plasmid for 48 h and treated with DMSO (vehicle) or with 5 µM β‐lap‐P1 together with 8 µM β‐lap. Samples were similarly prepared as described in β‐lap‐binding proteins and were subjected to immunoblotting under reducing conditions using the indicated antibodies.

### 5.12 Vimentin‐β‐Lap Binding Site Mapping

For identification of β‐lap binding sites on vimentin by MS, purified human vimentin (0.5 mg/ml) was incubated with β‐lap‐P1 for 2 h at room temperature. Protein‐ligand complexes were subjected to tryptic digestion and LC‐MS/MS analysis. The assignment of fragment ions in the MS/MS of the modified and unmodified peptides was obtained with the SEQUEST software tool “FuzzyIons” (distributed by ThermoFinnigan) to annotate the spectra from β‐lap‐P1‐treated and DMSO‐treated samples. This analysis verifies both the peptide amino acid sequence and the location and mass of the modification (+379.15 Da).

### 5.13 Vimentin Oxidation Analysis by Mass Spectrometry

Purified human vimentin (1 mg/ml) was incubated with DMSO or an equivalent amount of β‐lap. The protein samples were mixed with 5 × SDS loading buffer (Yeasen, no DTT) before SDS gel electrophoresis. The gels were stained for 30 min with a solution of Coomassie Brilliant Blue R‐250 and decolorized using water, followed by MS analysis.

### 5.14 Molecular Docking

The antiparallel tetramer structure of vimentin was generated using the HADDOCK2.4 web server (https://wenmr.science.uu.nl/haddock2.4/) [34, 70] with restraints derived from the photo‐crosslinking sites identified by MS. Two vimentin dimers were used as input: Dimer 1 (PDB: 3SWK)(containing residue 197) was derived from the crystal structure of the human vimentin coil 1B fragment, and Dimer 2 (containing residue 329) was derived from the crystal structure of the vimentin coil 2 fragment (PDB: 3KLT), which encompasses residues 261–335. The docking restraint applied for generating the antiparallel tetramer was specified as: “assign (segid A and resid 197) (segid B and resid 329) 10 5 5”, enforcing a distance restraint between residue 197 of one dimer and residue 329 of the opposing dimer to guide antiparallel alignment. The antiparallel tetramer satisfying this restraint was selected for subsequent docking with β‐lap.

Structure preparation, protonation, energy minimization, and molecular docking were performed using MOE (Molecular Operating Environment, version [2022.02], Chemical Computing Group). Prior to docking, the tetramer structure was prepared by repairing missing atoms and optimizing the hydrogen bond network via Protonate3D at default ionization states (pH 7.0). Water molecules were removed, and atomic restraint strength was set to 10 with a flat‐bottom restraint extent of 0.25 Å to prevent excessive positional deviations during minimization. Energy minimization was performed with a root‐mean‐square gradient of 0.1 kcal/mol/Å to optimize the conformation of the antiparallel tetramer.

For docking of β‐lap to vimentin, residues 197 and 329 were defined as the binding site. β‐lap was placed at the binding site using the Triangle Matcher placement method. Poses were generated and refined using the Amber10:EHT force field with the Induced Fit method, which allows sidechain flexibility upon ligand binding. The final complex structure was selected based on the lowest GBVI/WSA dG binding energy score and visual inspection of the binding pose consistency with experimental data. The final complex structure is provided in Figure 3H.

### 5.15 Surface Plasmon Resonance Assay

SPR data were acquired on Biacore 8K+/2583519 (Cytiva) at room temperature. CM5 Sensor Chip (Cytiva) was activated by using sulfo‐NHS/EDC chemistry in a buffer consisting of KCl, NaCl, and surfactant P20. The chip was subsequently immobilized with the vimentin/vimentin fragment at a concentration of about 0.5 mg/ml in TBS and reached target densities of 8000–13 000 resonance units. A 10 mM β‐lap stock in 100% DMSO was diluted 20‐fold into PBST (0.1% Tween) and further serially diluted with PBST containing 5% DMSO to the indicated concentrations. Data were analyzed with Biacore 8K+ Evaluation Software. Affinity and binding kinetic parameters were determined by fitting using the 1:1 interaction model.

### 5.16 Microscale Thermophoresis

MST was performed according to the detailed instructions for full affinity measurements using a Monolith NT.115 instrument (NanoTemper). A 16‑point 1:1 serial dilution of β‑lap was prepared in MST buffer (50 mM Tris‑HCl pH 7.4, 150 mM NaCl, 0.05% Tween‑20). Vimentin was mixed with each dilution, incubated for 120 min at room temperature, and loaded into standard capillaries. Thermophoresis was measured at 25°C with 20% LED power and medium MST power. Data were analyzed using MO. Affinity Analysis software (NanoTemper); the Kd was calculated by fitting the normalized fluorescence to a dose‑response curve. Each measurement was performed in triplicate with a signal‑to‑noise ratio > 5.

### 5.17 Recombinant Protein Expression and Purification

The cDNA encoding full‐length human vimentin (UniProt ID: P08670) was cloned into the pET‐28a vector (Novagen), which appends an N‐terminal His_6_ tag to the recombinant protein. Plasmids expressing fragments and mutants of vimentin protein were transformed into BL21(DE3) *E. coli* competent cells by heat shock and induced overnight with 1 mM IPTG at OD_600_ = 0.8. Cell pellets were collected by centrifugation (4000 g, 15 min), resuspended in 40 mL lysis buffer (50 mM Tris–HCl, 150 mM NaCl, 0.2 mM PMSF, pH 7.4), and lysed using sonication for 30 min (2 s on, 2 s off). Cellular debris was removed by centrifugation (20 min, 15 000 g). Recombinant proteins were purified on a pre‐equilibrated Ni‐chelating column, washed with washing buffer (50 mM Tris–HCl, 150 mM NaCl, 10 mM imidazole, pH 7.4) and eluted with eluting buffer (50 mM Tris–HCl, 150 mM NaCl, 250 mM imidazole, pH 7.4). Purified proteins were concentrated in a centrifugal ultrafiltration device (3 kDa cut‐off) and gel‐filtered on an AKTA Pure 25 M system using a Superdex 75 10/300 GL column in Tris buffered saline (50 mM Tris–HCl, 150 mM NaCl, pH 7.4). The peak fractions were collected and concentrated to 2 mg/mL and stored at 4°C. Full‐length vimentin protein was purified under urea‐denatured conditions with similar procedures. 8 M urea was added in all the buffers when purifying full‐length vimentin and removed by a step‐wise dialysis into Tris‐buffered saline with 6, 4, 2, and finally 0 M urea. Site‐directed mutagenesis was performed to generate E197A, E329A, K334A, D367A, and C328S mutations using the QuikChange protocol. All constructs were verified by DNA sequencing.

### 5.18 Histological Analysis

Mice were euthanized and the tissues were surgically resected, and tumors together with the indicated organs (liver, kidney, spleen, heart and lung) were collected without perfusion. Tissue fixation, paraffin embedding, sectioning and vimentin immunohistochemical staining were performed as previously described [13]. Whole‐slide images were acquired with a Panoramic SCAN scanner (3DHISTECH, Budapest, Hungary), and vimentin positivity was quantified in Image J (NIH) as the area fraction of positive signal per field, with three randomly selected fields per mouse (*n* = 2 mice per group).

### 5.19 Quantitative Reverse‐Transcription PCR

Cellular RNA was isolated using TRNzol (TIANGEN) and converted to cDNA using the High‐Capacity cDNA Reverse Transcription Kit (Yeasen). Gene expression was assessed using the 2× SYBR Green Kit (Yeasen) according to the manufacturer's instructions.

### 5.20 Confocal Microscopy

Cells were seeded into 4‐well glass coverslips (1 × 10^4^ cells/well). After cells were cultured overnight, the culture medium was replaced with the indicated treatments for the specified durations. After incubation, the cells were treated with indicated antibody for 30–50 min and then rinsed three times with cold PBS. Fluorescence images were recorded on a Leica TCS SP8 STED confocal microscope (Germany).

### 5.21 Pharmacokinetic Analysis

Pharmacokinetics of sustained β‐lap were evaluated in male Sprague Dawley rats (*n* = 3 per group). Rats received CD‐β‐lap intravenously at 10 mg/kg or β‐lap suspension orally at doses of 100 or 200 mg/kg (β‐lap molar‐equivalent dose). Serial blood samples (0.15 mL) were collected at 2, 5, 10, 15, 30 min and at 1, 2, 4, 6, 10, 24, 48 h post‐dosing, and were centrifuged (4°C, 7000 rpm) for 3 min to collect the plasma. Samples were then deproteinated and centrifuged at 13 000 rpm for 10 min. Supernatants were harvested, and the plasma β‐lap concentrations were quantified by LC‐MS/MS (AB SCIEX QTRAP 5500).

### 5.22 The Sustained β‑Lap Formulation

The sustained β‑lap formulation used for in vivo studies was a crystalline suspension. It was prepared as a crystalline suspension in vehicle (0.5% carboxymethylcellulose sodium + 0.1% Poloxamer 188 in saline). Owing to the intrinsically low aqueous solubility of β‑lap crystals, the dissolution rate of the drug from the suspension was limited, resulting in a slow and sustained release profile following administration. Consequently, this formulation provided a prolonged low‑concentration exposure in vivo, recapitulating the sustained low‐concentration treatment paradigm in vitro.

### 5.23 In Vivo Anti‐Tumor Efficacy

To evaluate the in vivo anti‐tumor effect of sustained β‐lap, we established cell‐derived xenograft (CDX) models. Briefly, KPC cells were isolated from tumors of *LSL‐Kras^G12D/^* ^+^ *; LSL‐Trp53^R172H/^* ^+^ *; Pdx‐1‐Cre* (KPC) genetically engineered pancreatic cancer mice. Female BALB/c nude mice (6 weeks old, 18–22 g; Vital River, Beijing, China) received subcutaneous flank injections of 2.0 × 10^6^ KPC cells, 1.0 × 10^6^ MIA Paca‐2 cells, 5×10^6^ NCI‐N87 or A549 cells for CDX establishment.

Tumor volume was calculated as (length × width^2^)/2. When the tumor volume reached ∼50 to 100 mm^3^, KPC cell‐bearing mice were randomized (*n* = 4 per group) to receive saline or sustained β‐lap. β‐lap was administered 100 mg/kg once a day from day 0 to day 25. BALB/c nude mice were subcutaneously inoculated with 1 × 10^6^ MIA Paca‐2 cells. Mice bearing MIA Paca‐2 tumors were randomized into three groups (*n* = 5): (i) saline; (ii) β‐lap 100 mg/kg orally twice a day; and (iii) gemcitabine 50 mg/kg intravenously twice weekly. The treatments lasted for 4 weeks. For the NCI‐N87 model, mice received saline (control), paclitaxel (15 mg/kg, intravenously once weekly), or the sustained β‐lap formulation (100 mg/kg, orally twice daily). The treatments lasted for 34 days. For the A549 model, mice received saline (control), docetaxel (10 mg/kg, intravenously once weekly), or the sustained β‐lap formulation (300 mg/kg, orally once daily), and the treatments lasted for 25 days. The difference in β‐lap dosage reflects the distinct tolerated doses across xenograft models (established in pilot tolerability studies). Tumor size and body weight were measured every 2–3 days. At the end of the experiment, 24 h post the last administration, mice were euthanized, and tumors were surgically resected, washed in PBS, weighed, and photographed. Tumor weights were compared by the Mann–Whitney *U*‐test. Animals were euthanized if the tumor burden reached a humane endpoint or if adverse effects or body weight loss, as defined by IACUC, occurred.

### 5.24 Humane Endpoints

Mice were euthanized when tumors reached a maximum volume of 1500 mm^3^, or if they exhibited >20% body weight loss, severe ulceration, necrosis, or signs of distress such as lethargy, hunched posture, or difficulty in ambulation. Any animals meeting the humane endpoint criteria were euthanized immediately by CO_2_ asphyxiation followed by cervical dislocation.

### 5.25 Quantification and Statistical Analysis

Data are presented as the mean ± SEM throughout. Exact n values and significance symbols are defined in each figure legend. For two‐group comparisons with adequate sample sizes (*n* > 3) or non‐normally distributed data, the two‐sided Mann–Whitney *U*‐test was used. Western blot quantified from independent replicates was analyzed with the two‐tailed unpaired Student's *t*‐test. Matched factorial comparisons were analyzed by two‐way ANOVA followed by Šídák's multiple‐comparisons test. Data distributions were examined by Q–Q plots. Correlations were assessed by linear regression, and the coefficient of determination with the corresponding *p* value was reported. All statistical analyses were performed using GraphPad Prism (version 9.0; GraphPad Software, San Diego, CA, USA). Image quantification was performed with Image J (NIH).

## Author Contributions

**Lei Dou**: investigation, methodology, data curation, formal analysis, validation, writing of the original draft. **Huiqin Liu**: formal analysis, supervision, reviewing and editing. **Kaixin Wang**: investigation, methodology, data curation, validation, reviewing and editing. **Yi Xia**: investigation, methodology, data curation, formal analysis. **Zhidong Chen**: methodology, data curation. **Feng Qian**: conceptualization, funding acquisition, project administration, supervision, reviewing and editing.

## Funding

This work was supported by grants from the Beijing Frontier Research Center for Biological Structure (Grant No. 041500002), and National Natural Science Foundation of China (Project No. 82473854, 82607645).

## Ethics Statement

All animal procedures were approved by the Animal Ethics Committee of Tsinghua University (IACUC; approval No. 16‐QF1).

## Conflicts of Interest

The authors declare no conflicts of interest.

## Supporting information




**Supporting File 1**: advs78747‐sup‐0001‐SuppMat.pdf.

**Supporting File 2**: advs78747‐sup‐0002‐SuppMat.docx.

## Data Availability

The data that support the findings of this study are available from the corresponding author upon reasonable request.
