# Supplementary material for: Pharmacokinetic Tuning Redirects β‐Lapachone From NQO1 Bioactivation to Vimentin‐Targeted Oxidation in Cancer
Source: Adv Sci (Weinh). 2026 Nov 9:e78747. Online ahead of print. doi: 10.1002/advs.78747 (PMC13748893; doi:10.1002/advs.78747)
Supplement: Supplementary file 1 [file ADVS-9999-e78747-s001.pdf]

Pharmacokinetic Tuning Redirects β-Lapachone from NQO1 Bioactivation to Vimentin-Targeted Oxidation in Cancer

Lei Dou[1+], Huiqin Liu[1+], Kaixin Wang[1], Yi Xia[1], Zhidong Chen[1], Feng Qian[1*]

[1]*School of Pharmaceutical Sciences, Beijing Frontier Research Center for Biological Structure, and Key Laboratory of Bioorganic Phosphorus Chemistry & Chemical Biology (Ministry of Education), Tsinghua University, Beijing 100084, P.R. China.*

[*]To whom correspondence should be addressed:

Email: qianfeng@tsinghua.edu.cn

+: These authors contributed equally to this work.

Supporting Information

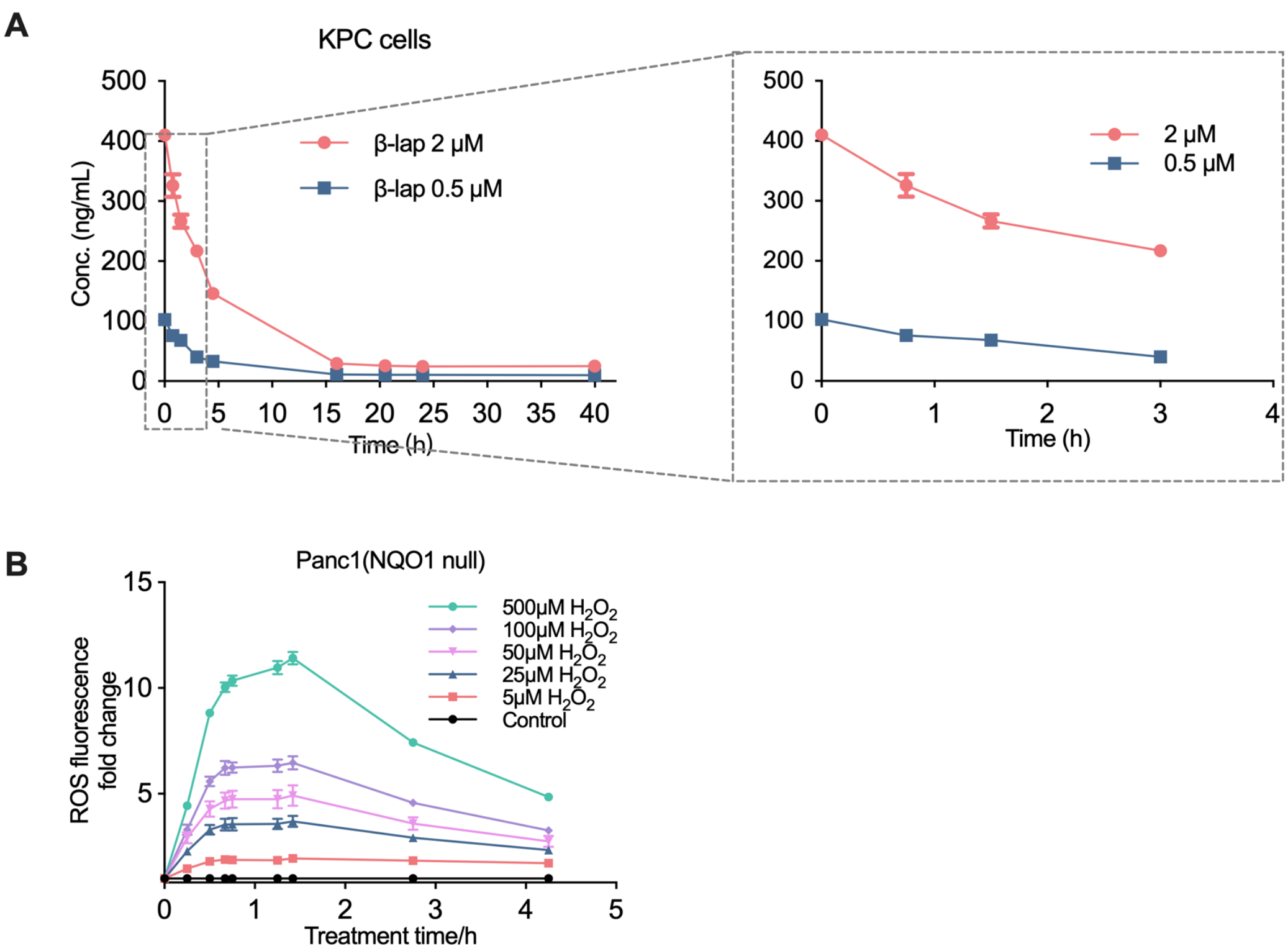

Fig. S1 Drug exposure profile of β-lap in KPC cells. A, Quantification of β-lap in culture medium by mass spectrometry following treatment of KPC cells with 2 µM (red) or 0.5 µM (blue) β-lap over 40 hours. The inset shows an enlarged view of the first 3 h of the left panel, showing the rapid initial decline phase. Concentration (Conc.) is expressed in ng/mL. **B**, The ROS levels were examined by DCFDA fluorescence at different time after hydrogen peroxide treatment in NQO1 null Panc1 cells. n = 3.

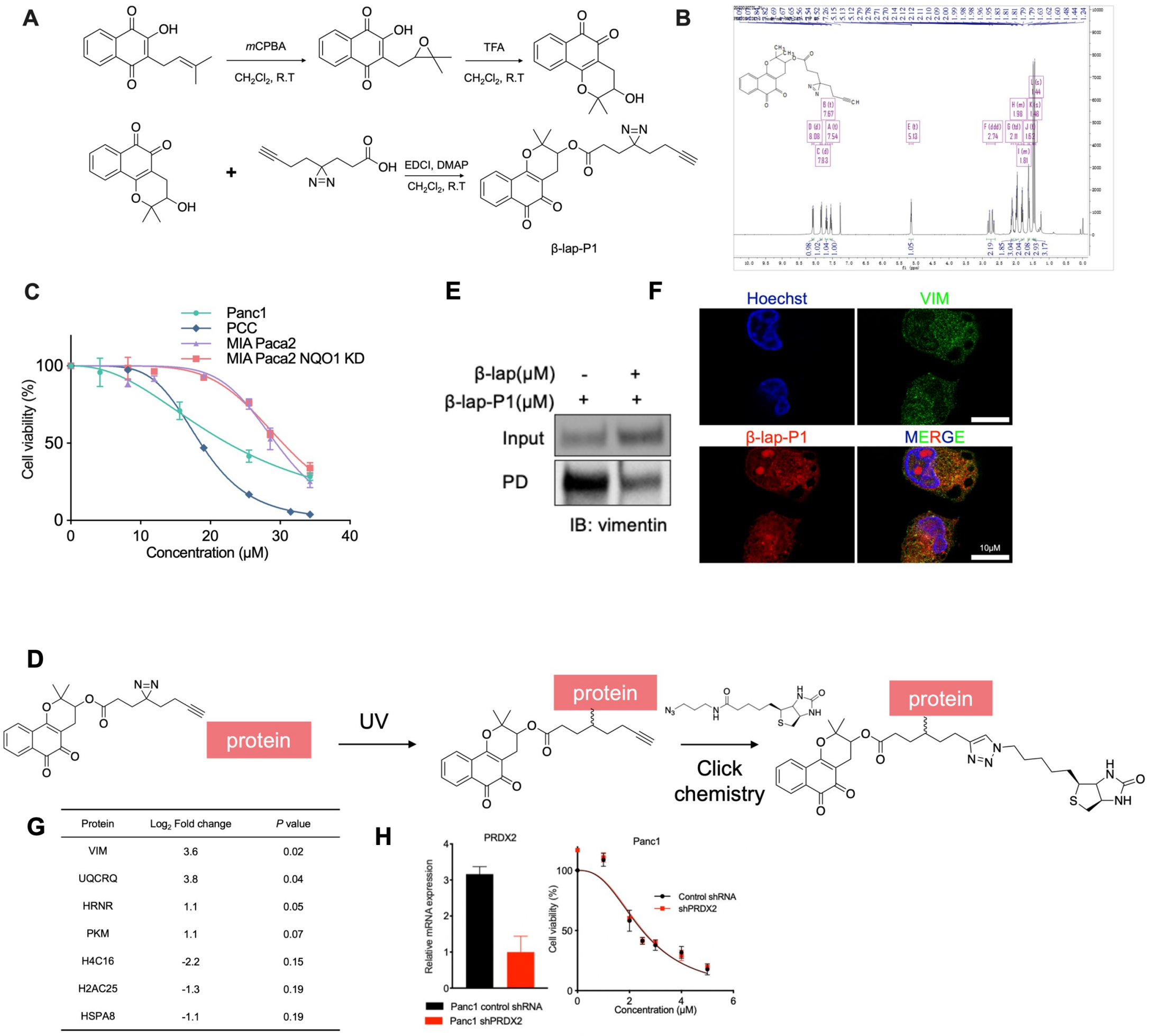

Protein	Log_2 Fold change	*P* value
VIM	3.6	0.02
UQCRQ	3.8	0.04
HRNR	1.1	0.05
PKM	1.1	0.07
H4C16	-2.2	0.15
H2AC25	-1.3	0.19
HSPA8	-1.1	0.19

Fig. S2 Identification and validation of vimentin as a direct binding target of β-lap. **A,** Synthesis of photoactive β-lap probes (β-lap-P1). **B**, NMR data of β-lap-P1. **C**, Survival assays of β-lap-P1 on Panc1 cells, MIA Paca-2 cells, MIA Paca-2 NQO1 KD cells and KPC cells exposed to β-lap-P1 (treatment B). **D**, A scheme that depicts the reactions to form the β-lap-P1 and proteins conjugates. **E**, β-lap binds vimentin in Panc1 cells. Panc1 cells were incubated with β-lap-P1 or with β-lap-P1+β-lap for 4 h. After UV-induced labeling, cell lysates were conjugated with biotin and proteins were purified over Streptavidin affinity beads. Then they were subjected to SDS-PAGE and western blotted with anti-vimentin antibody. The bottom panel represents streptavidin-HRP detection of endogenous biotinylated species used as a loading control. **F**, Colocalization analysis of VIM and β-lap-P1 in Panc1 cells. Panc1 cells were treated with β-lap-P1 for 4 h, permeabilized with Triton X-100, and incubated with 1 mM TCEP, 102 μM TBTA, 1 mM $CuSO_4$, and 100 μM TAMRA-N_3 for 1 h at room temperature for

click labeling, followed by immunostaining with anti-VIM antibody (green). **G,** Ranked analysis of the β-lap-P1 competition pulldown experiment. n = 3. **H**, Relative mRNA expression of PRDX2 in Panc1 cells transfected with control shRNA or PRDX2-specific shRNA, determined by qPCR(left). Cell viability of Panc1 cells after PRDX2 knockdown and treatment with β-lap (treatment B) (right). n = 3.

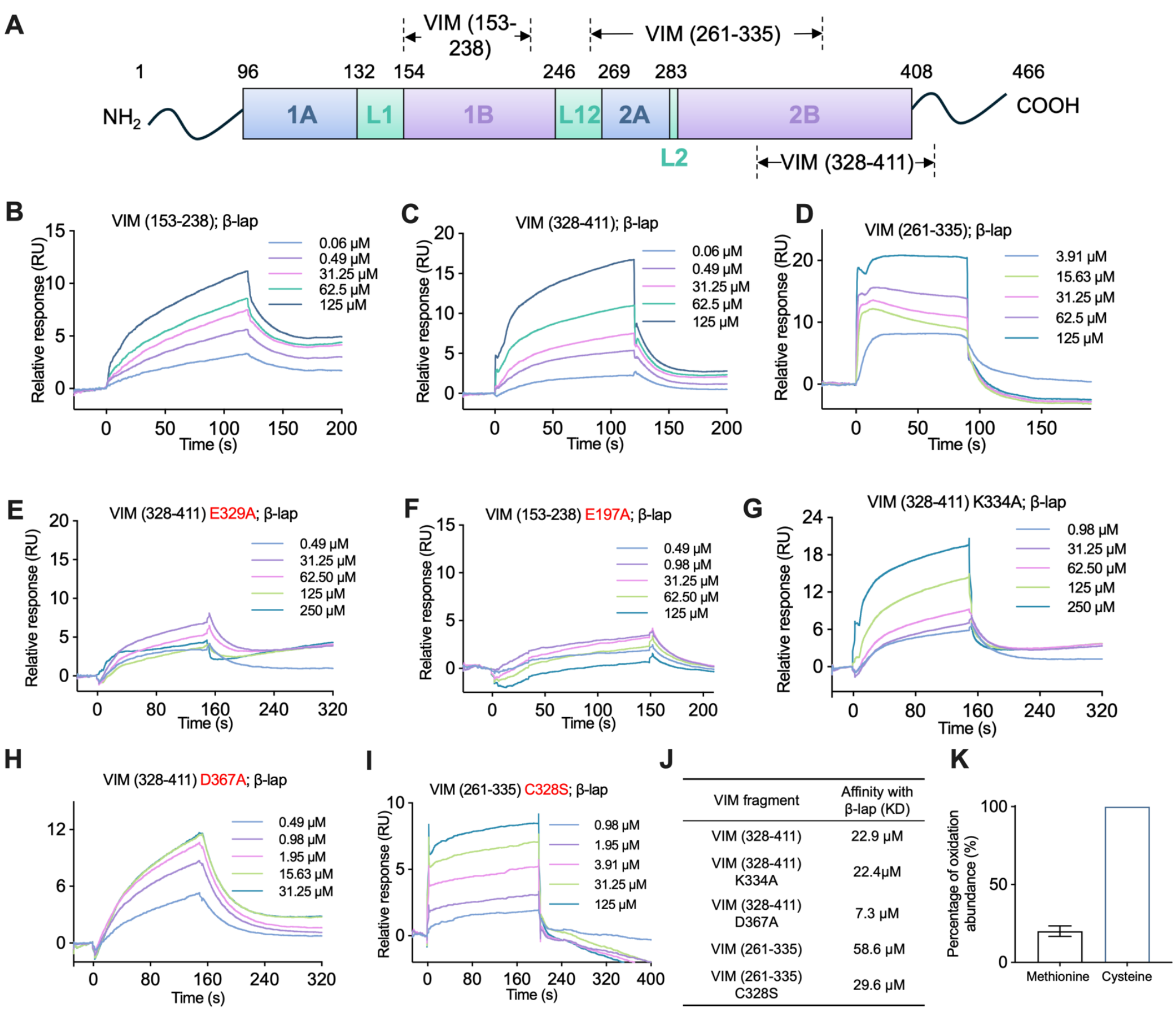

Fig. S3 Mapping the β-lap-binding site on vimentin. A, Schematic of the molecular structure of the vimentin monomer including the various segments and linker domains, as well as the head and tail domain. **B,** In SPR assays, VIM (153-238), VIM (328-411) (**C**) and VIM (261-335) (**D**) were incubated with β-lap at the indicated concentrations. **E-I**, In SPR assays, VIM (153-238), VIM (261-335) and VIM (328-411) different mutants were incubated with β-lap at the indicated concentrations. The summarized affinity data are presented in (**J**). **K**, Quantification of oxidative modifications at C328 and methionine residues by LC-MS/MS.

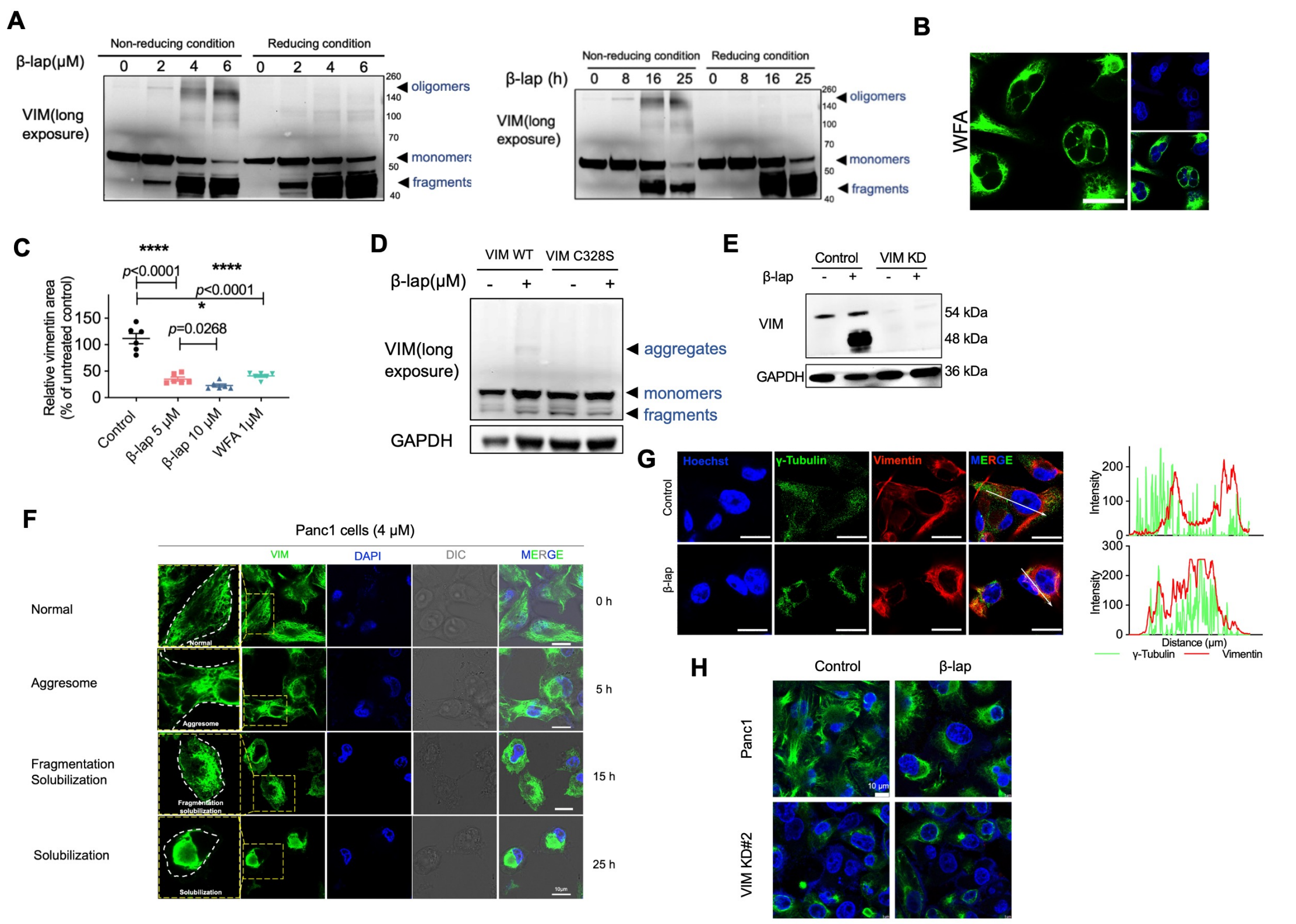

Fig. S4 β-lap-induced vimentin aggregates and fragments. A, Aggregation and cleavage of vimentin were increased in Panc1 cells after β-lap treatment B for 24 h in a dose- and time-dependent way by western blotting. **B**, Maximum intensity projections from confocal imaging of vimentin (VIM) immunostained Panc1 after treatment with WFA. (Scale bar, 20 μm.) Quantification is shown in (**C**). n = 6. **D,** Western blotting of VIM in VIM KD cells re-expressing WT VIM or the C328S mutant after vehicle or β-lap (treatment B). E, Western blotting of VIM levels in WT Panc1 and VIM KD (KD#2) cells treated with and without β-lap (treatment B). **F**, Confocal microscopy of vimentin (green) using IF staining on Panc1 after various treatment durations with β-lap. Scale bars, 20 μm. Insets show individual cells with outlined contours (white dashed lines). DIC, differential interference contrast. **G**, Assessment of aggresome formation. Cells were treated with DMSO or β-lap, stained with Hoechst (blue), vimentin (red) and γ-Tubulin (green). Scale bars, 20 μm. Fluorescence intensity was plotted along the arrows. Quantification is shown in the left panel. Data were analyzed by two-tailed unpaired *t*-test. **H**, WT Panc1 or VIM KD Panc1(KD#2) were treated with DMSO or β-lap

visualized by confocal microscopy. Minimal residual VIM was detected in VIM knockdown cells and remained unchanged upon β-lap treatment. Scale bars, 10 μm.

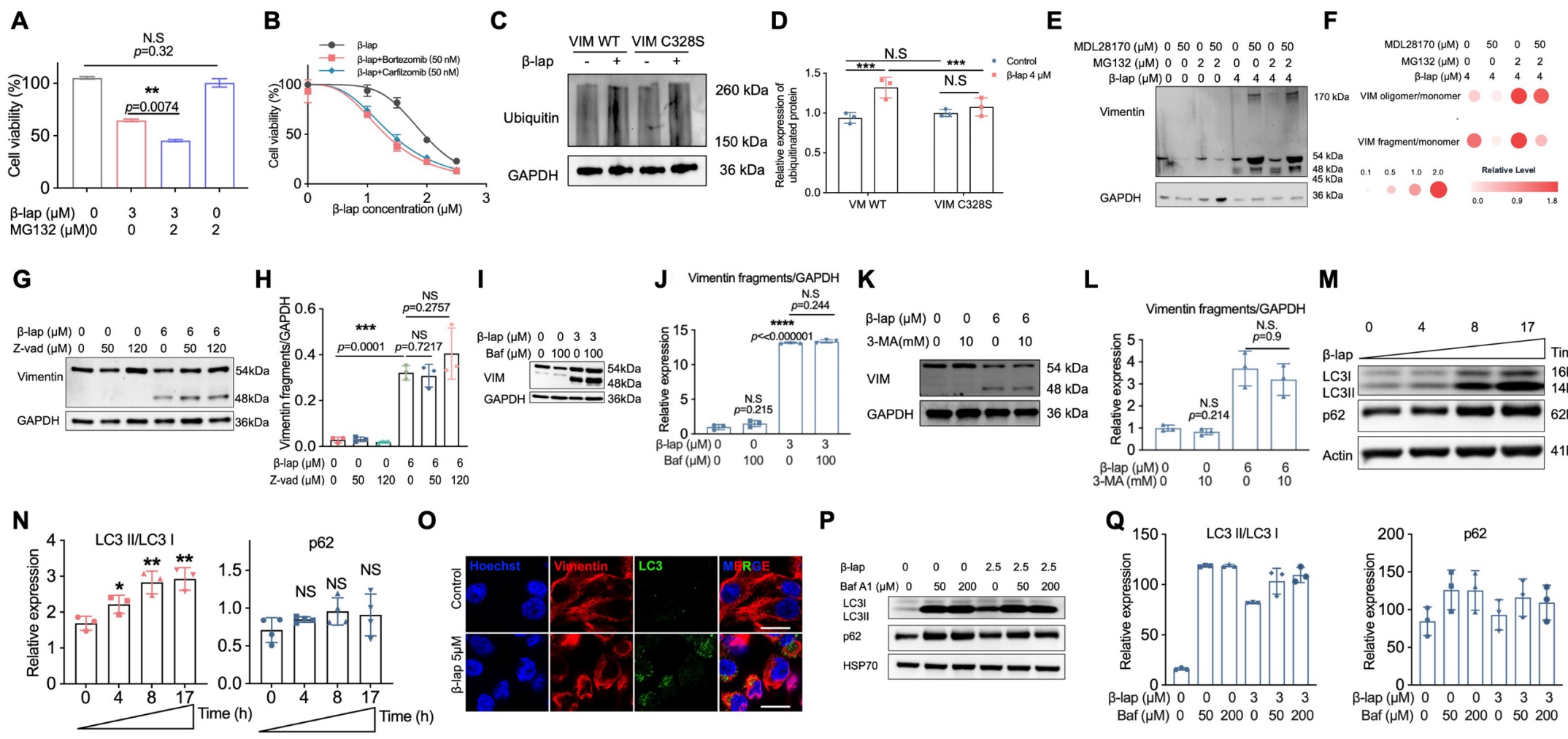

Fig. S5 β-lap-induced vimentin aggregates and fragments drove proteostasis loss. **A-B**, Panc1 cells were cotreated with β-lap and MG132 (**A**) or β-lap and bortezomib/carfilzomib (**B**), and cell viability was assessed after 24 h. The 0 μM β-lap groups in the β-lap + bortezomib/carfilzomib combination treatments served as inhibitor alone controls (cell viability ~93.3% and ~95.3%, respectively). **C**, Western blotting of ubiquitinated proteins in WT Panc1 and C328S-expressing cells treated with vehicle or β-lap (4 μM). Quantification is shown in (**D**). **E**, Western blotting of VIM monomers, aggregates and fragments in Panc1 cells treated with β-lap alone, β-lap plus MG132, β-lap plus MDL-28170, MG132 alone or MDL-28170 alone as indicated. Quantification is shown in (**F**) and the levels of VIM oligomers and degradation products were normalized to the full-length monomer band in each lane. Vimentin monomers and fragments after β-lap and Z-VAD-FMK treatment (**G**) or Bafilomycin (**I**) or 3-MA (**K**) by western blotting. Quantification is shown in (**H**), (**J**), and (**L**). **M**, Protein levels of LC3II, LC3I, p62, were examined by western blotting after various treatment durations with β-lap. Quantification is shown in (**N**). **O**, Panc1 cells were treated with DMSO or β-lap and stained with Hoechst (blue), vimentin (red) and LC3 (green). Scale bars, 20 μm. **P**, Protein levels of LC3II, LC3I, p62, were examined by western blotting at different time points after β-lap and bafilomycin treatment.

Quantification is shown in (**Q**). Data were analyzed by two-tailed unpaired *t*-test (A, H, J, L, N, Q) and two-way ANOVA with Šídák's multiple-comparisons test (**D**). n = 3.

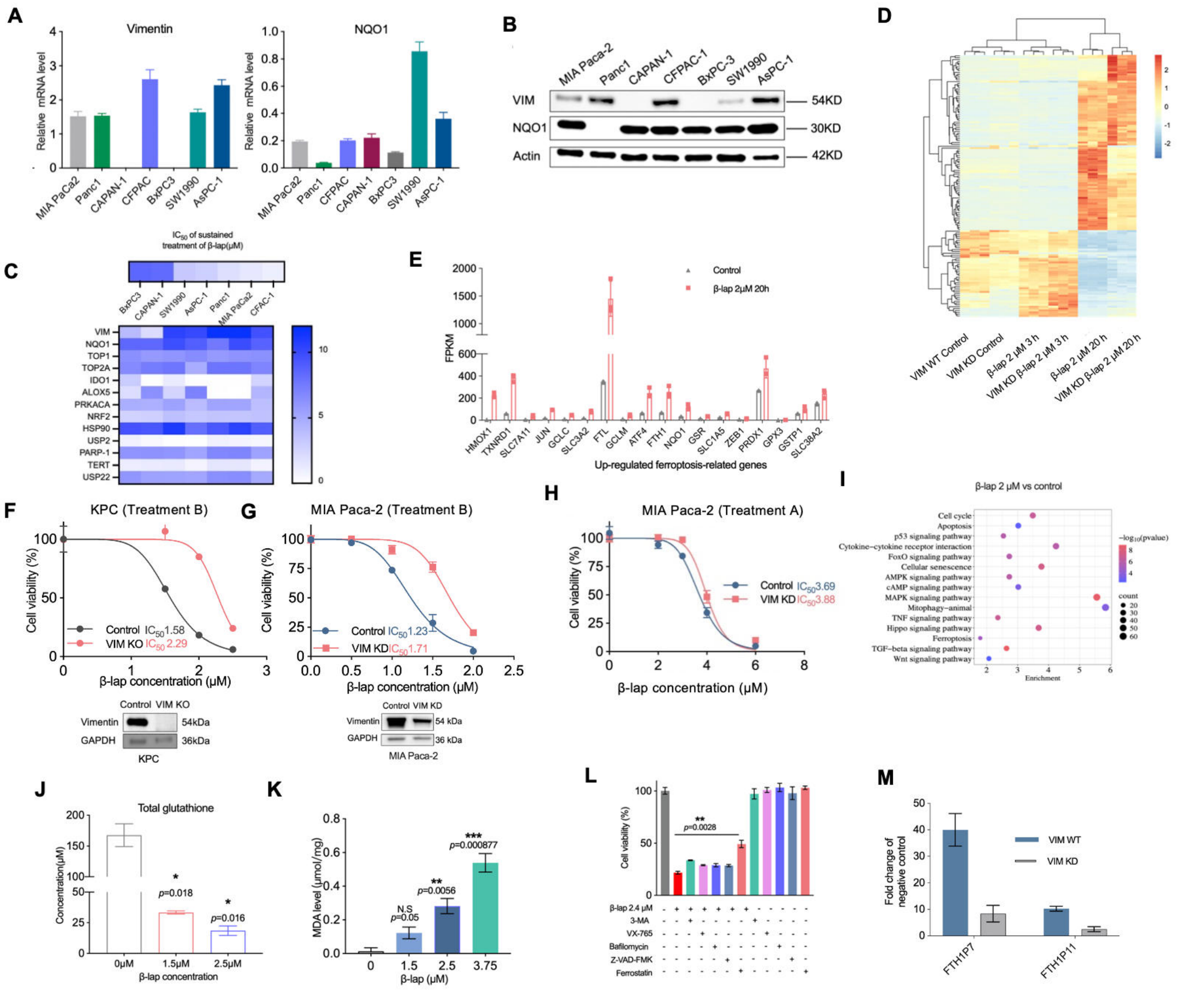

Fig. S6 Vimentin was essential for sustained β-lap-mediated ferroptosis. A, Relative mRNA levels of VIM and NQO1 in seven pancreatic cancer cell lines. **B**, Western blotting of NQO1, VIM in seven pancreatic cancer cell lines. **C**, IC_{50} values of β-lap (treatment B) in the indicated cell lines are shown in the top row. Expression levels of previously reported β-lap targets across the indicated cell lines. The expression of these reported targets did not correlate with β-lap sensitivity under sustained exposure except VIM. **D**, Heatmap of β-lap-treated (3 h/20 h) samples in WT Panc1 cells compared to VIM knockdown cells. **E**, Upregulated genes in RNA-seq associated with ferroptosis were shown. **F**, Survival assays of VIM-wildtype (control) and VIM-knockout KPC cells exposed to β-lap (treatment B). **G**, Survival assays of vimentin wildtype and knockdown MIA Paca-2 cells exposed to β-lap (treatment B) or (**H**) β-lap (treatment A). **I**, Bubble plot of enriched pathways in β-lap-treated (20 h) samples compared to WT. The x-axis shows the GeneRatio (the ratio of input genes annotated in a term), and the y-axis lists the top enriched GO terms. **J**, The levels of GSH and (**K**) MDA were tested after β-lap (treatment B) for 24 h. **L**, Panc1 cells were exposed to β-

lap (treatment B) for 24 h alone or with Z-VAD-FMK (7 μM), 3-MA (1.5 mM), VX-765 (3 μM), Bafilomycin (2 nM), Ferrostatin-1 (3 μM). After 24 h incubation, the cell viability was measured. **M**, mRNA levels of FTH1P7 and FTH1P11 in wildtype and VIM knockdown cells treated with β-lap (treatment B) in RNA-seq data. Data represent fold change over untreated controls. n = 3. Data were analyzed by two-tailed unpaired *t*-test.

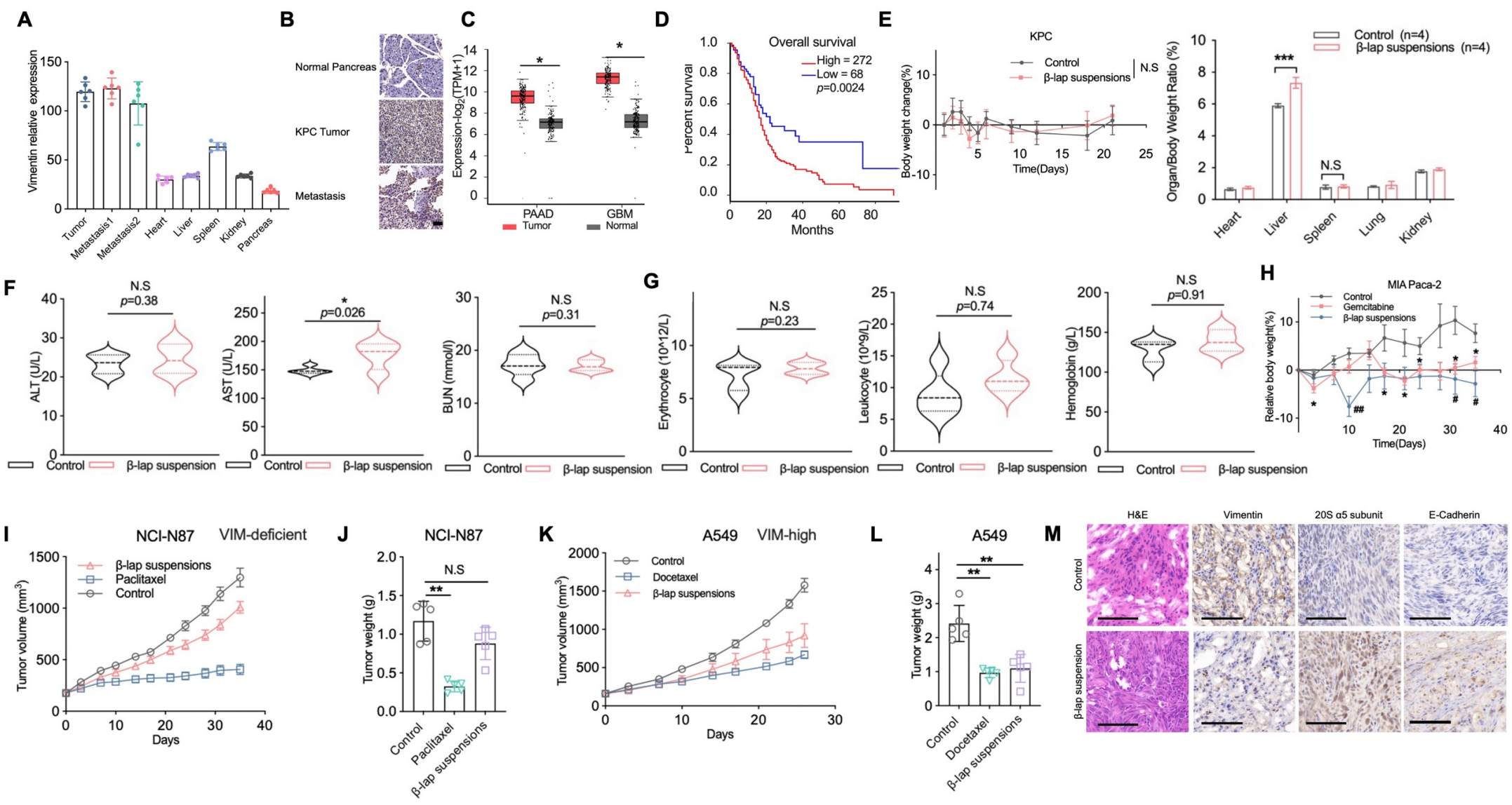

Fig. S7 Sustained low-concentration β-lap decreased tumor burdens in mouse tumor models with high VIM expression. A, Expression of vimentin is elevated in PDAC tumors in the KPC mice and metastasis lesions compared with adjacent normal areas analyzed from IHC staining of indicated tissues. For each tissue, 3 randomly selected microscopic fields per mouse were quantified from n = 2 mice (6 fields in total), and each dot represents one microscopic field. **B**, Vimentin staining of the normal pancreas and KPC tumors and metastasis from KPC mice. Scale bars, 50 μm. **C**, Protein expression levels of vimentin in tumor sites and normal tissues in PAAD and GBM in TCGA datasets. **D**, The Kaplan-Meier overall survival of patients of PAAD and GBM in the TCGA dataset that was stratified by vimentin high vs vimentin low (split by the 20th percentile). *P* values were determined by the log-rank (Mantel–Cox) test. **E**, Body weight changes and the organ to body weight ratio of KPC cell-bearing mice were shown. n = 4. **F**, ALT, AST and BUN levels of KPC cell-bearing mice after sustained β-lap 100 mg/kg. ALT, Alanine Aminotransferase; AST, Aspartate transaminase; BUN,

Blood Urea Nitrogen. n = 4. **G**, The hematological assessments of KPC cell bearing mice after β-lap administration. n = 4. **H**, Body weight changes of MIA Paca-2 tumor-bearing mice were shown. n = 5. $^{*}P < 0.05$ and $^{**}P < 0.01$ control vs first-line therapy; $^{\#}$P < 0.05 and $^{\#\#}$P < 0.01 control vs sustained β-lap group. **I,** NCI-N87 tumor-bearing mice were treated with saline, paclitaxel (15 mg/kg, intravenously once weekly), or the sustained β-lap (100 mg/kg, orally twice daily) and tumor growth curves were shown. **J,** Tumor weight changes of the mice models in (**I**) were shown. **K,** A549 tumor-bearing mice were treated with saline, docetaxel (10 mg/kg, intravenously once weekly), or sustained β-lap (300 mg/kg, orally once daily) and tumor growth curves were shown. **L,** Tumor weight changes of the mice models in (**K**) were shown. (n = 5 mice per group in I–L.). **M**, Representative H&E or IHC staining of vimentin, 20S α5 subunit and E-Cadherin in tumor tissues in KPC tumor-bearing mice treated with saline or sustained β-lap 100 mg/kg. Data were analyzed by two-tailed unpaired *t*-test (C, E, F, G and H). Tumor weights in (J and L) were analyzed by the Mann–Whitney *U*-test.

Fig. S8 LC-MS/MS identification of β-lap binding sites on vimentin and oxidative modifications.

A, Recombinant vimentin was incubated with β-lap-P1, and peptides carrying a +379.154 Da mass shift (probe adduct) were identified.

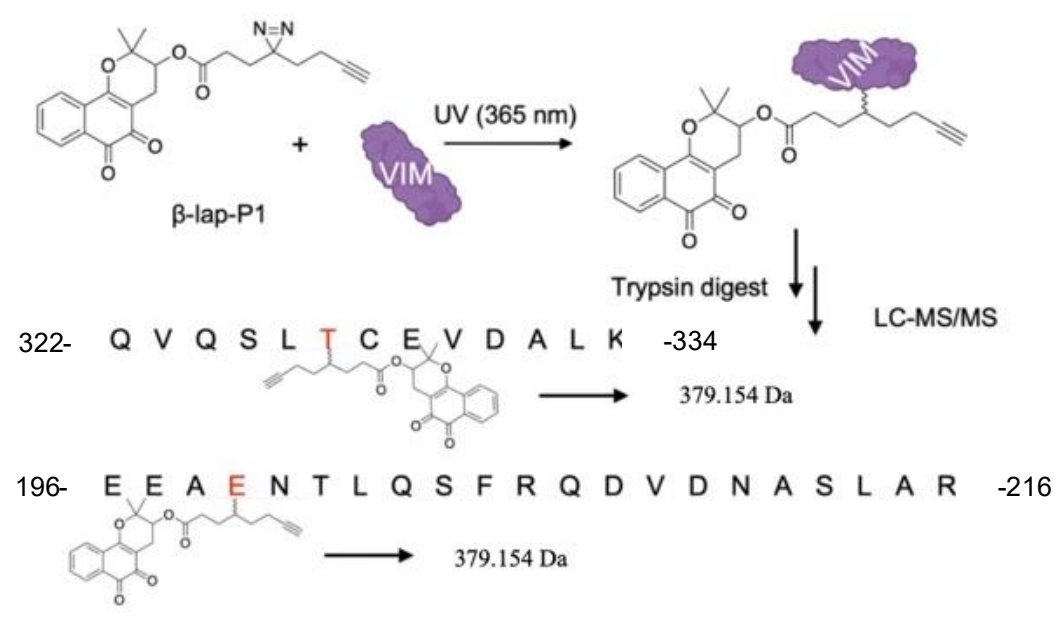

Peptide Summary

Sequence: EEAENTLQSFRQDVDNASLAR E1-C23H23O5 (379.15400 Da), E2-C23H23O5 (379.15400 Da), E4-C23H23O5 (379.15400 Da)
Charge: +5, Monoisotopic m/z: 706.92212 Da (-2.66 mmu/-3.76 ppm), MH+: 3530.58149 Da, RT: 101.8279 min,
Identified with: Sequest HT (v1.17); XCorr:3.84,
Fragment match tolerance used for search: 0.02 Da
Fragments used for search: y-H2O; y-NH3; b; b-H2O; b-NH3; y

Fragment Matches

Value Type: Theo. Mass [Da]

Ion Series | Neutral Losses | Precursor Ions | Internal Fragments

#1	b+	b2+	b3+	b4+	b5+	Seq.	y+	y2+	y3+	y4+	y5+	#2
1	509.20387	255.10557	170.40614	128.05642	102.64660	E-C23H23...						21
2	1017.40046	509.20387	339.80501	255.10557	204.28591	E-C23H23...	3022.39818	1511.70273	1008.13758	756.35500	605.28546	20
3	1088.43758	544.72243	363.48404	272.86485	218.49334	A	2514.20158	1257.60443	838.73871	629.30585	503.64614	19
4	1596.63417	798.82072	532.88291	399.91400	320.13266	E-C23H23...	2443.16447	1222.08587	815.05967	611.54657	489.43872	18
5	1710.67710	855.84219	570.89722	428.42473	342.94124	N	1934.96788	967.98758	645.66081	484.49743	387.79940	17
6	1811.72478	906.36603	604.57978	453.68665	363.15078	T	1820.92495	910.96611	607.64650	455.98669	364.99081	16
7	1924.80884	962.90806	642.27446	481.95767	385.76759	L	1719.87727	860.44227	573.96394	430.72478	344.78128	15
8	2052.86742	1026.93735	684.96066	513.97231	411.37930	Q	1606.79321	803.90024	536.26925	402.45376	322.16446	14
9	2139.89944	1070.45336	713.97133	535.73032	428.78571	S	1478.73463	739.87095	493.58306	370.43911	296.55275	13
10	2286.96786	1143.98757	762.99414	572.49742	458.19939	F	1391.70260	696.35494	464.57238	348.68111	279.14634	12
11	2443.06897	1222.03812	815.02784	611.52270	489.41962	R	1244.63419	622.82073	415.54958	311.91400	249.73266	11
12	2571.12755	1286.06741	857.71403	643.53734	515.03133	Q	1088.53308	544.77018	363.51588	272.88873	218.51244	10
13	2686.15449	1343.58088	896.05635	672.29408	538.03672	D	960.47450	480.74089	320.82968	240.87408	192.90072	9
14	2785.22290	1393.11509	929.07915	697.06118	557.85040	V	845.44756	423.22742	282.48737	212.11735	169.89533	8
15	2900.24985	1450.62856	967.42147	725.81792	580.85579	D	746.37914	373.69321	249.46457	187.35024	150.08165	7
16	3014.29277	1507.65003	1005.43578	754.32865	603.66438	N	631.35220	316.17974	211.12225	158.59351	127.07626	6
17	3085.32989	1543.16858	1029.11481	772.08793	617.87180	A	517.30927	259.15827	173.10794	130.08278	104.26768	5
18	3172.36192	1586.68460	1058.12549	793.84594	635.27820	S	446.27216	223.63972	149.42890	112.32350	90.06025	4
19	3285.44598	1643.22663	1095.82018	822.11695	657.89502	L	359.24013	180.12370	120.41823	90.56549	72.65385	3
20	3356.48309	1678.74519	1119.49922	839.87623	672.10244	A	246.15607	123.58167	82.72354	62.29447	50.03703	2
21						R	175.11895	88.06311	59.04450	44.53520	35.82961	1

Fragment Spectrum

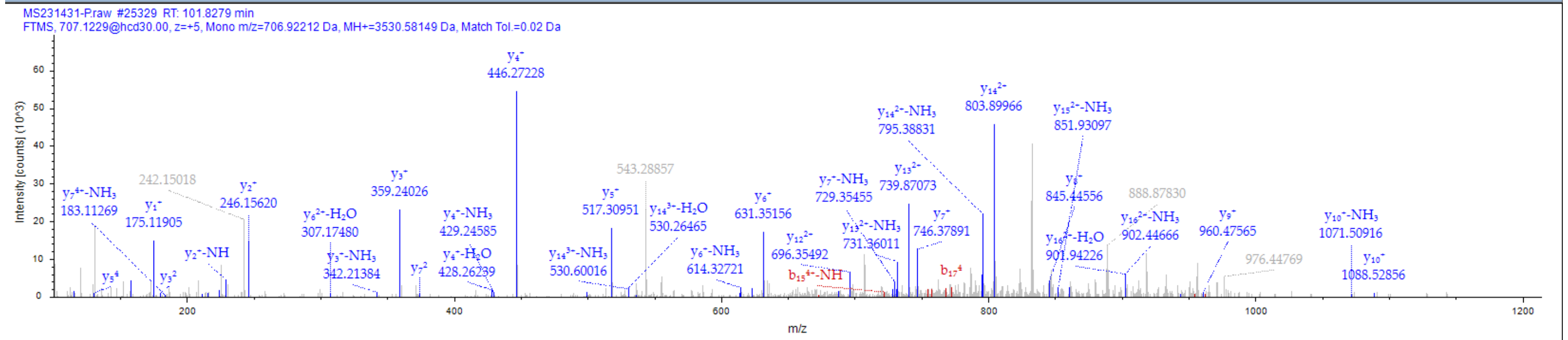

Peptide Summary

Sequence: QVQSLTCEVDALK, C7-Carbamidomethyl (57.02146 Da), S4-C23H23O5 (379.15400 Da), T6-C23H23O5 (379.15400 Da)
Charge: +3, Monoisotopic m/z: 750.35706 Da (-1.1 mmu/-1.47 ppm), MH+: 2249.05661 Da, RT: 99.6849 min,
Identified with: Sequest HT (v1.17); XCorr:3.03,
Fragment match tolerance used for search: 0.02 Da
Fragments used for search: y-H_2O; y-NH_3; b; b-H_2O; b-NH_3; y

Fragment Matches

Value Type: Theo. Mass [Da]

Ion Series | Neutral Losses | Precursor Ions | Internal Fragments

#1	b^+	b^{2+}	b^{3+}	Seq.	y^+	y^{2+}	y^{3+}	#2
1	129.06585	65.03657	43.69347	Q				13
2	228.13427	114.57077	76.71627	V	2121.00134	1061.00431	707.67197	12
3	356.19285	178.60006	119.40247	Q	2021.93293	1011.47010	674.64916	11
4	822.37887	411.69308	274.79781	S-C23H23...	1893.87435	947.44081	631.96297	10
5	935.46294	468.23511	312.49250	L	1427.68832	714.34780	476.56763	9
6	1415.66462	708.33595	472.55972	T-C23H23...	1314.60426	657.80577	438.87294	8
7	1575.69526	788.35127	525.90327	C-Carbami...	834.40258	417.70493	278.80571	7
8	1704.73786	852.87257	568.91747	E	674.37193	337.68960	225.46216	6
9	1803.80627	902.40677	601.94027	V	545.32934	273.16831	182.44796	5
10	1918.83321	959.92025	640.28259	D	446.26092	223.63410	149.42516	4
11	1989.87033	995.43880	663.96163	A	331.23398	166.12063	111.08285	3
12	2102.95439	1051.98083	701.65632	L	260.19687	130.60207	87.40381	2
13				K	147.11280	74.06004	49.70912	1

Fragment Spectrum

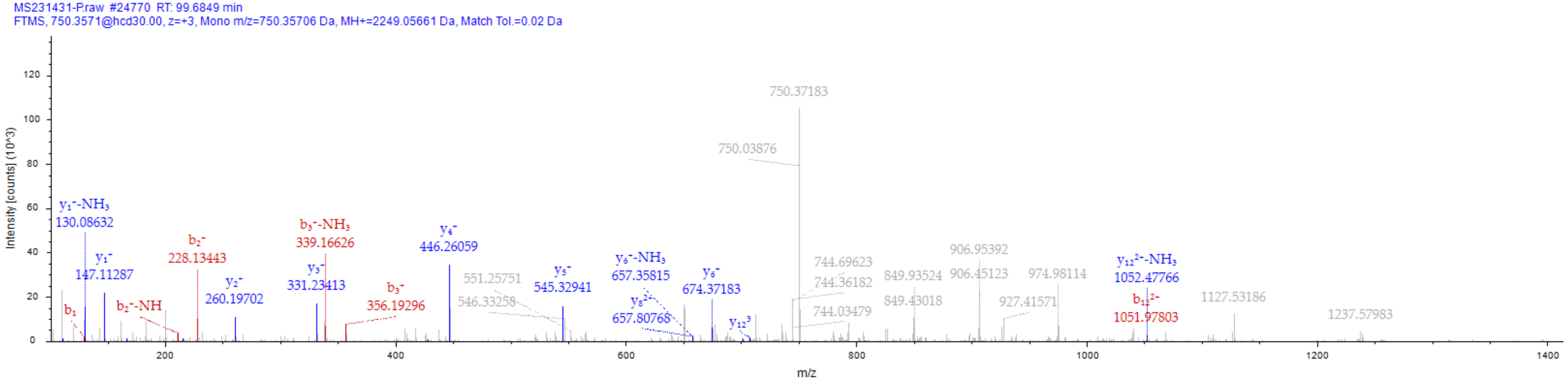

B, Recombinant vimentin was incubated with β-lap and subjected to LC-MS/MS analysis. The spectra reveal a +32 Da mass shift at residue C328 (indicative of oxidation).

Peptide Summary

Sequence: QVQSLTCEVDALKGTNESLER, C7-Dioxidation (31.98983 Da)
Charge: +3, Monoisotopic m/z: 784.71539 Da (-1.04 mmu/-1.33 ppm), MH+: 2352.13163 Da, RT: 37.5854 min,
Identified with: Sequest HT (v1.17); XCorr:4.00,
Fragment match tolerance used for search: 0.02 Da
Fragments used for search: y-H_2O; y-NH_3; b; b-H_2O; b-NH_3; y

Fragment Matches

Value Type: Theo. Mass [Da]

Ion Series | Neutral Losses | Precursor Ions | Internal Fragments

#1	b⁺	b²⁺	b³⁺	Seq.	y⁺	y²⁺	y³⁺	#2
1	129.06585	65.03657	43.69347	Q				21
2	228.13427	114.57077	76.71627	V	2224.07617	1112.54172	742.03024	20
3	356.19285	178.60006	119.40247	Q	2125.00775	1063.00751	709.00744	19
4	443.22487	222.11608	148.41314	S	1996.94917	998.97823	666.32124	18
5	556.30894	278.65811	186.10783	L	1909.91715	955.46221	637.31057	17
6	657.35662	329.18195	219.79039	T	1796.83308	898.92018	599.61588	16
7	792.35563	396.68145	264.79006	C-Dioxidati...	1695.78540	848.39634	565.93332	15
8	921.39822	461.20275	307.80426	E	1560.78639	780.89683	520.93365	14
9	1020.46664	510.73696	340.82706	V	1431.74380	716.37554	477.91945	13
10	1135.49358	568.25043	379.16938	D	1332.67538	666.84133	444.89665	12
11	1206.53069	603.76898	402.84842	A	1217.64844	609.32786	406.55433	11
12	1319.61476	660.31102	440.54310	L	1146.61133	573.80930	382.87529	10
13	1447.70972	724.35850	483.24142	K	1033.52726	517.26727	345.18061	9
14	1504.73118	752.86923	502.24858	G	905.43230	453.21979	302.48228	8
15	1605.77886	803.39307	535.93114	T	848.41084	424.70906	283.47513	7
16	1719.82179	860.41453	573.94545	N	747.36316	374.18522	249.79257	6
17	1848.86438	924.93583	616.95965	E	633.32023	317.16375	211.77826	5
18	1935.89641	968.45184	645.97032	S	504.27764	252.64246	168.76406	4
19	2048.98047	1024.99388	683.66501	L	417.24561	209.12644	139.75339	3
20	2178.02307	1089.51517	726.67921	E	304.16155	152.58441	102.05870	2
21				R	175.11895	88.06311	59.04450	1

Fragment Spectrum

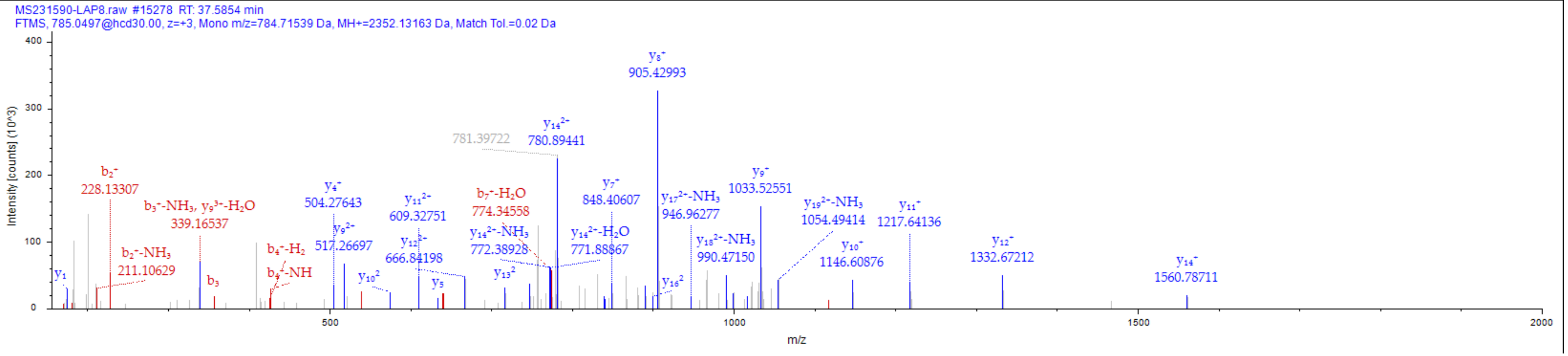

C, Recombinant vimentin was incubated with β-lap and subjected to LC-MS/MS analysis. The spectra reveal a +48 Da mass shift at residue C328 (indicative of oxidation).

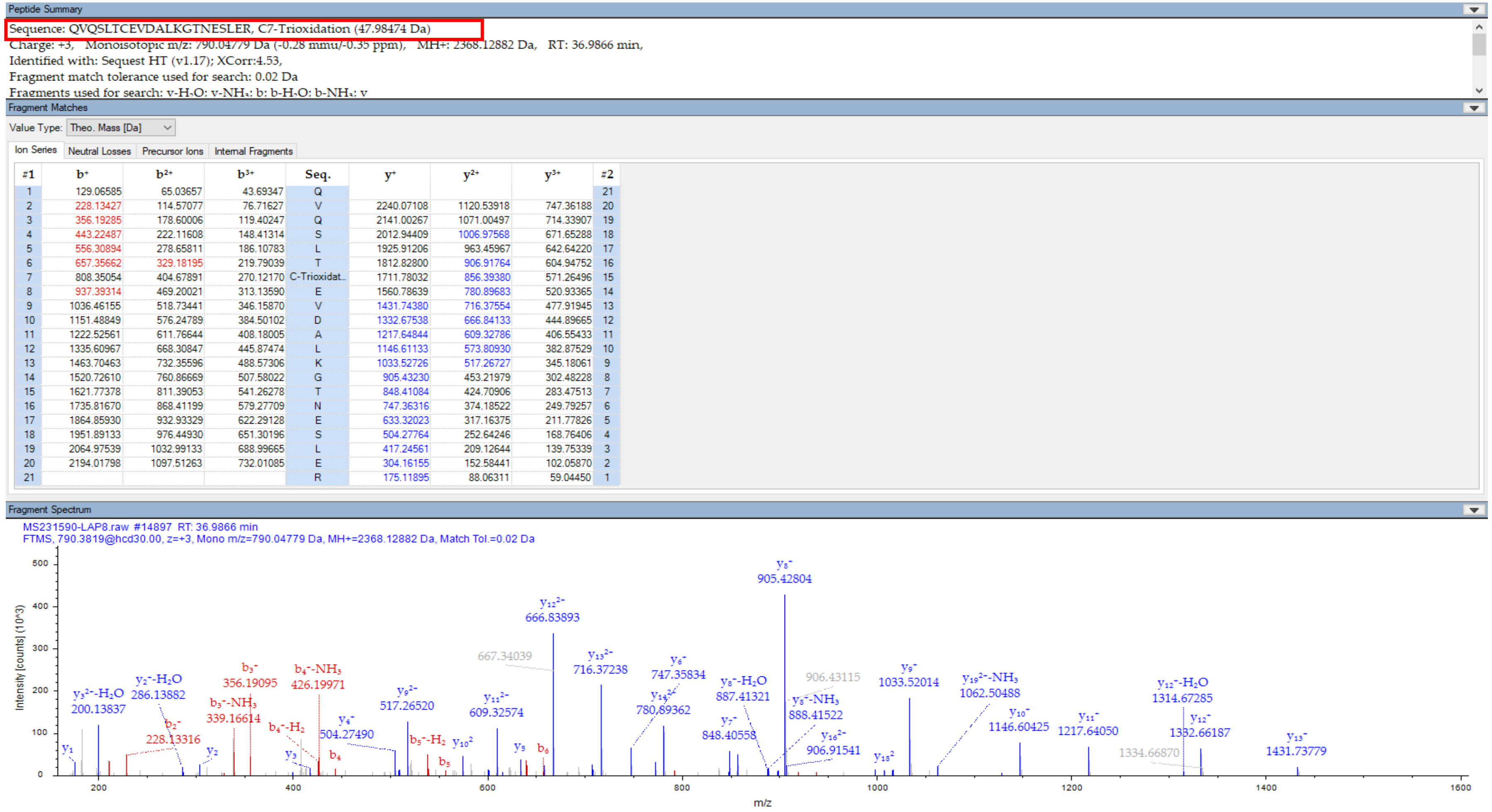

#1	b⁺	b²⁺	b³⁺	Seq.	y⁺	y²⁺	y³⁺	#2
1	129.06585	65.03657	43.69347	Q				21
2	228.13427	114.57077	76.71627	V	2240.07108	1120.53918	747.36188	20
3	356.19285	178.60006	119.40247	Q	2141.00267	1071.00497	714.33907	19
4	443.22487	222.11608	148.41314	S	2012.94409	1006.97568	671.65288	18
5	556.30894	278.65811	186.10783	L	1925.91206	963.45967	642.64220	17
6	657.35662	329.18195	219.79039	T	1812.82800	906.91764	604.94752	16
7	808.35054	404.67891	270.12170	C-Trioxidat...	1711.78032	856.39380	571.26496	15
8	937.39314	469.20021	313.13590	E	1560.78639	780.89683	520.93365	14
9	1036.46155	518.73441	346.15870	V	1431.74380	716.37554	477.91945	13
10	1151.48849	576.24789	384.50102	D	1332.67538	666.84133	444.89665	12
11	1222.52561	611.76644	408.18005	A	1217.64844	609.32786	406.55433	11
12	1335.60967	668.30847	445.87474	L	1146.61133	573.80930	382.87529	10
13	1463.70463	732.35596	488.57306	K	1033.52726	517.26727	345.18061	9
14	1520.72610	760.86669	507.58022	G	905.43230	453.21979	302.48228	8
15	1621.77378	811.39053	541.26278	T	848.41084	424.70906	283.47513	7
16	1735.81670	868.41199	579.27709	N	747.36316	374.18522	249.79257	6
17	1864.85930	932.93329	622.29128	E	633.32023	317.16375	211.77826	5
18	1951.89133	976.44930	651.30196	S	504.27764	252.64246	168.76406	4
19	2064.97539	1032.99133	688.99665	L	417.24561	209.12644	139.75339	3
20	2194.01798	1097.51263	732.01085	E	304.16155	152.58441	102.05870	2
21				R	175.11895	88.06311	59.04450	1
