# Supplementary material for: Pharmacokinetic Tuning Redirects β‐Lapachone From NQO1 Bioactivation to Vimentin‐Targeted Oxidation in Cancer
Source: Adv Sci (Weinh). 2026 Nov 9:e78747. Online ahead of print. doi: 10.1002/advs.78747 (PMC13748893; doi:10.1002/advs.78747)
Supplement: Supplementary file 2 [file ADVS-9999-e78747-s002.docx]

**SUPPLEMENTAL TABLES**

**Table S1. Primers used for RT-qPCR analysis, related to Methods.**

| **Names** | **Sequences (5’->3’)** |
| --- | --- |
| *NQO1* Forward Primer | ATGTATGACAAAGGACCCTTCC |
| *NQO1* Reverse Primer | TCCCTTGCAGAGAGTACATGG |
| *VIM* Forward Primer | GTCTCTGGCACGTCTTGAC |
| *VIM* Reverse Primer | TGCTGTTCCTGAATCTGAGC |
| *GAPDH* Forward Primer  *GAPDH* Reverse Primer | CATGTACGTTGCTATCCAGGC  CTCCTTAATGTCACGCACGAT |

**Table S2. Primers for shRNA and siRNA, related to Methods.**

| **Names** | **Sequences (5’->3’)** |
| --- | --- |
| *NQO1*-shRNA | CCGGCATGTTATCAAATCTGGGTATCTCGAGATACCCAGATTTGATAACATGTTTTT |
| *Non-silencing (NS) scrambled shRNA* | CCTAAGGTTAAGTCGCCCTCG |
| *siVIM* | GCAGAAGAAUGGUACAAA UTT  AUUUGUACCAUUCUUCUGC TT |

**Table S3. Primers for sgRNA, related to Methods.**

| **Names** | **Sequences (5’->3’)** |
| --- | --- |
| *VIM* A03  *VIM* A04  *VIM* H01  *VIM* H02  *NQO1* NH5  *NQO1* NH6  *NQO1* NA1  *NQO1* NA3 | CTTGACCTTGAACGCAAAGTGG  AGATTCCACTTTGCGTTCAAGG  GGATTCCACTTTCCGTTCAAGG  GGAAACGTCCACATCGATCTGG  AGGATACTGAAAGTTCGCAGGG  CACAATATCTGGGCTCAGATGG  CAGAAGAGCACTGATCGTACTGG  TAAGCCAGAACAGACTCGGCAGG |

**Table S4. Antibodies, chemicals, peptides, and recombinant proteins, critical commercial assays and recombinant DNA related to Methods.**

| Reagent or Resource | Source | Identifier |
| --- | --- | --- |
| Antibodies | | |
| Anti-vimentin (V9) | Thermo Fisher Scientific | 14-9897-82 |
| Anti-NQO1 | Proteintech | Cat# 11451-1-AP |
| Anti-LC3B | Cell Signaling Technology | Cat# 3868 |
| Anti-p62/SQSTM1 | Abcam | Cat# ab109012 |
| Anti-E-cadherin | Cell Signaling Technology | Cat# 3195 |
| Anti-Proteasome 20S alpha + beta antibody | Abcam | Cat# ab22673 |
| Anti-HSP70 | Cell Signaling | Cat# 4872 |
| HRP-streptavidin | Thermo Fisher Scientific | Cat# N100 |
| HRP-goat anti-rabbit IgG | Thermo Fisher Scientific | Cat# 31460 |
| GAPDH | Proteintech | Cat# 60004-1-Ig |
| Gamma Tubulin Monoclonal Antibody | Thermo Fisher Scientific | Cat# MA5-32514 |
| Chemicals, Peptides, and Recombinant Proteins | | |
| β-Lapachone (β-lap) | BioDuro | N/A |
| β-lap-P1 (photoaffinity probe) | This paper | N/A |
| Dicoumarol (DIC) | MedChemExpress | Cat# HY-N0645 |
| Withaferin A (WFA) | MedChemExpress | Cat# HY-N2065 |
| Bortezomib | MedChemExpress | Cat# HY-10227 |
| Carfilzomib | MedChemExpress | Cat# HY-10455 |
| MDL-28170 | MedChemExpress | Cat# HY-18236 |
| Z-VAD-FMK | Solarbio life sciences | Cat# IZ0810 |
| Bafilomycin A1 | Selleck Chemicals | Cat# S1413 |
| 3-Methyladenine (3-MA) | Selleck Chemicals | Cat# S2767 |
| Ferrostatin-1 | MedChemExpress | Cat# HY-100579 |
| Lipofectamine 3000 | Thermo Fisher Scientific | Cat# L3000015 |
| P3000 reagent | Thermo Fisher Scientific | Cat# L3000015 |
| DynabeadsTM M-280 | Thermo Fisher Scientific | Cat# 11205D |
| Critical commercial assays | | |
| DCFDA-Cellular ROS Detection Assay Kit | Abcam | Cat# ab113851 |
| Proteostat Aggresome Detection Kit | Enzo Life Sciences | Cat# ENZ-51035 |
| RNAsimple Total RNA Extraction Kit | TIANGEN | Cat# DP419 |
| NovoScript@Plus All-in-one 1st Strand cDNA Synthesis SuperMix | Novoprotein | Cat# E047 |
| Recombinant DNA | | |
| GFP-VIM (wildtype) | This paper | N/A |
| VIM-OE plasmid | This paper | N/A |
| Software and Algorithms | | |
| GraphPad Prism (v9.0) | GraphPad Software | https://www.graphpad.com/ |
| ImageJ (Fiji) | NIH | https://imagej.net/software/fiji/ |
| Biacore 8K+ Evaluation Software | Cytiva | N/A |
| MO.Affinity Analysis | NanoTemper | N/A |
| FlowJo (v10) | BD Biosciences | https://www.flowjo.com/ |
